# Crystal structures of eight mono-methyl alkanes (C_26_–C_32_) *via* single-crystal and powder diffraction and DFT-D optimization

**DOI:** 10.1107/S2052252515010271

**Published:** 2015-07-05

**Authors:** Lee Brooks, Michela Brunelli, Philip Pattison, Graeme R. Jones, Andrew Fitch

**Affiliations:** a ESRF, CS 40220, 38043 Grenoble CEDEX 9, France; b School of Physical and Geographical Sciences, Lennard–Jones Laboratories, Keele University, Staffordshire ST5 5BG, UK; c ILL, CS 20156, 38042 Grenoble CEDEX 9, France; d SNBL/ESRF, CS 40220, 38043 Grenoble CEDEX 9, France; e Crystallography Competence Centre, EPFL, 1015 Lausanne, Switzerland

**Keywords:** mono-methyl alkanes, powder diffraction, synchrotron radiation, DFT-D, single-crystal diffraction, insect pheromones

## Abstract

The crystal structures of eight mono-methyl alkanes have been determined from single-crystal or high-resolution powder X-ray diffraction using synchrotron radiation. Mono-methyl alkanes can be found on the cuticles of insects and are believed to act as recognition pheromones in some social species, *e.g.* ants, wasps *etc*. The molecules were synthesized as pure *S* enantiomers and are (*S*)-9-methylpentacosane, C_26_H_54_; (*S*)-9-methylheptacosane and (*S*)-11-methylheptacosane, C_28_H_58_; (*S*)-7-methylnonacosane, (*S*)-9-methylnonacosane, (*S*)-11-methylnonacosane and (*S*)-13-methylnonacosane, C_30_H_62_; and (*S*)-9-methylhentriacontane, C_32_H_66._ All crystallize in space group *P*2_1_. Depending on the position of the methyl group on the carbon chain, two packing schemes are observed, in which the molecules pack together hexagonally as linear rods with terminal and side methyl groups clustering to form distinct motifs. Carbon-chain torsion angles deviate by less than 10° from the fully extended conformation, but with one packing form showing greater curvature than the other near the position of the methyl side group. The crystal structures are optimized by dispersion-corrected DFT calculations, because of the difficulties in refining accurate structural parameters from powder diffraction data from relatively poorly crystalline materials.

## Abstract 2

The crystal structures of eight mono-methyl alkanes are solved from powder or single-crystal X-ray diffraction data, and optimized *via* DFT-D calculations.

## 1. Introduction   

The outer cuticle of an insect is covered with a layer of waxy organic molecules with carbon chain lengths up to around 45 atoms which can include *n*-alkanes, mono-, di-, tri- and tetramethyl alkanes, alkenes, dienes, also oxygen-containing compounds such as alcohols, esters, fatty acids, ketones, aldehydes *etc*. For example, Bonavita-Cougourdan *et al.* (1996 ▸) identified cholesterol and well over 50 hydrocarbons on the cuticle of slave ants *Formica cunicularia*, while Bagnères & Morgan (1991 ▸) confirmed some 26 hydrocarbon compounds on the cuticle of the ants *Manica rubida*. This layer acts as a barrier to dehydration (Lockey, 1988 ▸; Gibbs, 2002 ▸) but some of the molecules can also play a role as recognition pheromones, which allow social insects to distinguish their nest mates from insects of the same species from another nest, or from related species (*e.g.* Bagnères *et al.*, 1996 ▸; Cuvillier-Hot *et al.*, 2001 ▸; Dani *et al.*, 2001 ▸; Dapporto *et al.*, 2006 ▸; Lahav *et al.*, 1999 ▸; Ruther *et al.*, 2002 ▸; Singer, 1998 ▸; Zanetti *et al.*, 2001 ▸). It is the overall mixture of active compounds – a chemical signature – that allows specific insects to be identified. In some insects the cuticular hydrocarbon molecules can also be used to distinguish sex, age and social status.

Dani *et al.* (2001 ▸) noted that dousing paper wasps with *n*-alkanes had no apparent effect on kin recognition, whereas methyl-branched alkanes induced an aggressive response. Martin & Drijfhout (2009 ▸) comment that the wide range and large quantities of mono-methyl alkanes present on insects would suggest that they act as general indicators and it is the smaller quantities of alkenes and dimethylalkanes that are used to communicate the more complex information of nest-mate recognition. The differences in chemical properties between *n*-alkanes and methyl-branched alkanes are small, implying that stereochemical factors play a role, *e.g.* chirality, position of the methyl group(s) on the carbon chain or favourable conformations of the molecule allowing interaction with the insect’s receptors.

Energy-minimization calculations (Goodman, 1997 ▸) suggest that isolated *n*-alkane molecules adopt a folded conformation once the length of the chain is greater than 17 C atoms long because the stabilizing van der Waals interactions between the —CH_2_— groups in the folded chain outweigh the energy required to twist the chain from the preferred extended conformation. In crystalline *n*-alkanes, folding occurs with longer chains of the order of 150 C atoms and above with the tendency to fold into an integral number of pleats, thus excluding chain-end imperfections from the interior of the molecular packing (Ungar *et al.*, 1985 ▸).

The crystal structures of methyl-branched alkanes have received less attention and knowledge of their crystal structures, molecular packing and preferred conformations is unclear. The symmetric molecules 20-methylnonatriacontane (C_19_H_39_)_2_CH(CH_3_) and 18-methylpentatriacontane (C_17_H_35_)_2_CH(CH_3_), which have the methyl group attached to the central C atom of the main chain, were described with an extended chain conformation (Yamamoto *et al.*, 2004 ▸; Ikedou *et al.*, 2005 ▸), whereas melt- or solution-crystallization of the much longer C_96_H_193_CH(CH_3_)C_94_H_189_ produced a once-folded conformation (Ungar & Zeng, 2001 ▸). The influence of the side-chain position on the packing and crystal structures of methyl alkanes has not been extensively studied.

As part of a more general investigation into the compounds found in the cuticular layers of social insects and their action as recognition pheromones, we synthesized a series of enantiopure mono-methyl alkanes, main chain C_25_–C_31_ with the methyl group at a variety of positions. Here we report their crystal structures, determined *via* high-resolution powder diffraction using synchrotron radiation, with one example investigated by single-crystal diffraction of limited range, obtained fortuitously by the appearance of crystals in the powder specimen. Though solved from the diffraction data by standard direct methods (for the single crystal) and direct-space minimization techniques (for the powders), the structures have been optimized by dispersion-corrected DFT calculations, because of the difficulties of refining accurate structures from powder diffraction data of limited quality and *d*-spacing extent. Two distinct packing arrangements are seen depending on the position of the methyl group on the carbon chain.

## 2. Experimental   

### 2.1. Samples   

The *S* enantiomorphs of eight mono-methyl alkanes were synthesized using pseudoephedrine as chiral auxiliary (Myers *et al.*, 1997 ▸), *i.e.* (*S*)-9-methylpentacosane, C_26_H_54_; (*S*)-9-methylheptacosane and (*S*)-11-methylheptacosane, C_28_H_58_; (*S*)-7-methylnonacosane, (*S*)-9-methylnonacosane, (*S*)-11-methylnonacosane and (*S*)-13-methylnonacosane, C_30_H_62_; and (*S*)-9-methylhentriacontane, C_32_H_66_. The samples were purified by column chromatography through silica using petroleum, hexane or petroleum ether as solvent, to yield soft, waxy, low-melting-point white solids.

### 2.2. Powder diffraction   

High-resolution powder X-ray diffraction patterns were measured at wavelengths near 0.8 Å using beamline ID31 (Fitch, 2004 ▸, 2007 ▸) at the ESRF, Grenoble. Samples were introduced into 1 mm diameter thin-walled borosilicate-glass capillaries, cooled to 100 K (or 80 K in one instance) with a nitrogen-gas blower and spun axially to improve powder averaging. There was no apparent radiation damage during the measurements. The samples were not all measured during the same experimental session, and measurements were repeated following the experience gained using a 2 d X-ray detector (see below). Hence the data used for the final analyses were not all recorded under exactly the same conditions, resulting in a range in the statistical quality of the data (judged by the *R*_exp_ values). Generally powder diffraction patterns were obtained up to 40° 2θ, and were recorded over a period of up to 3 h.

Because of concerns about the possibility of granularity effects with the waxy samples which could not be easily ground before being loaded into the capillaries, measurements were also made at 120 K with a wavelength of 0.82 Å using the Swiss–Norwegian beamline BM01A, equipped with a two-dimensional MAR-345 image-plate detector system, to assess the spottiness (or otherwise) of the Debye–Scherrer rings. Granularity and texture effects can lead to inaccurate peak intensities with high-resolution powder data and impede structural analysis, *e.g.* as seen in the study of oxanorbornane (Palin *et al.*, 2007 ▸). In addition, the samples were melted and recrystallized by shock freezing in the cold nitrogen stream to investigate possible effects on crystal randomization or the formation of new structural phases. In general, the powder rings showed that it was possible to obtain diffraction patterns not unduly affected by powder-averaging problems, but that this was still a risk. In particular, 7-methylnonacosane (7-MeC_29_H_59_) showed individual diffraction spots from within the powder, indicating that there were larger crystals present possibly suitable for a single-crystal measurement.

The high-resolution powder diffraction patterns could be indexed from the positions of 20 to 30 low-angle peaks using the singular value decomposition approach (Coelho, 2003 ▸) implemented in the *TOPAS* analysis suite (Bruker, 2008 ▸). All patterns were indexed with monoclinic cells, space group *P*2_1_, *Z* = 2. It was clear that the samples exhibited two types of unit cell, distinguished by different *b* axis lengths of around 7.15 and 4.93 Å, respectively.

Structure solutions were attempted from the powder data by direct-space methods, using simulated annealing and the program *DASH* (David *et al.*, 2006 ▸). An extended molecule was introduced into the unit cell and its position (2 degrees of freedom), orientation (3 degrees of freedom) and carbon-chain torsion angles (limited to ±5° from the staggered 180° conformation) were allowed to vary. Solutions were found for the three compounds with unit-cell *b* axis lengths of ≃7.15 Å. Following the solution of the structure of 7-methylnonacosane from the single-crystal pattern (see below), which indicates greater deviations from a straight molecule than imposed in the initial analysis, molecules with *b* cell axis lengths of around 4.93 Å were allowed greater freedom in their torsion angles. In this way structures were obtained for the remaining compounds.

Although the basic structures could be solved from the powder data, Rietveld (1969 ▸) refinements were hard to stabilize. Without extensive restraints on bond distances, angles and torsions, the molecules distorted from what was chemically acceptable. This is not surprising given the many degrees of freedom and the true information content of a diffraction pattern from a powder of relatively modest crystallinity and effective *d*-spacing range.

### 2.3. Single-crystal diffraction   

Two plate-shaped crystals of (*S*)-7-methylnonacosane, about 200 µm in diameter and 20 µm thick, were extracted from the specimen and mounted in oil using a standard 0.3 mm diameter cryoloop. Both were investigated using beamline BM01A, giving diffraction patterns of limited range. It was apparent that one of the crystals was twinned.

The single-crystal diffraction pattern was indexed in a monoclinic cell similar to that derived from the powder pattern (see Table 1 ▸). The data were integrated and scaled with the *CrysAlis* package. Given the wavelength and the composition, differences between Friedel pairs were negligible. The structure solution and refinement were made *via* *SIR*92 (Altomare *et al.*, 1994 ▸) and *CRYSTALS* (Betteridge *et al.*, 2003 ▸), using only isotropic atomic displacement parameters for the C atoms because of the limited extent of the data. The molecule shows a distinct deviation from linear near the methyl side group.

## 3. DFT calculations   

It is not unusual that a powder diffraction pattern does not allow an accurate refinement of the crystal and molecular structures, even though it may be good enough to solve the basic molecular packing scheme from a direct-space global-minimization approach exploiting the known molecular connectivity. In these circumstances verifying and completing the structural analysis by quantum-mechanical density functional theory calculations is an attractive option. The use of dispersion-corrected DFT has been described by van de Streek & Neumann (2010 ▸, 2014 ▸), where it served as an effective check on the accuracy of structures derived from single-crystal and powder-diffraction studies, respectively.

The DFT program *CASTEP* (Clark *et al.*, 2005 ▸) was used, as integrated into the Accelrys *Materials Studio* 6.1 package, using the PBE functional (Perdew *et al.*, 1996 ▸), plane-wave energy cut-off of 520 eV (as in van de Streek & Neumann, 2010 ▸), and including dispersion interactions *via* the TS scheme (Tkatchenko & Scheffler, 2009 ▸). The *Materials Studio* ‘ultrafine’ geometry optimization convergence criteria were adopted, with a final Cartesian displacement of less than 5 × 10^−4^ Å, a maximum force of 0.01 eV Å^−1^ and maximum energy difference of 5 × 10^−6^ eV per atom. The unit-cell parameters were fixed at the experimental values. Optimizing the crystal structure of 7-MeC_29_H_59_ starting from the refined single-crystal model led to a DFT-minimized arrangement in which the molecules differed by a root-mean-square distance of 0.019 Å (with a maximum deviation of 0.042 Å) for the C atoms when overlaid using *Mercury* (Macrae *et al.*, 2008 ▸). The close agreement between the experimental and DFT-minimized structures suggests the experimental crystal structure is accurate despite the limitations of the data.

The structures obtained from powder data are less accurate, so as a test the calculation for 7-MeC_29_H_59_ was repeated after straightening the molecule so that all C—C—C—C main-chain torsion angles were 180°. Starting from this configuration and optimizing following the same procedure produced a structure with slightly poorer agreement with the single-crystal structure with a r.m.s. distance of 0.054 Å and a maximum distance of 0.132 Å. This level of agreement is still better than the average r.m.s. deviation of 0.084 Å reported by van de Streek & Neumann (2010 ▸) for the 241 ordered molecular crystal structures they tested. The two DFT-minimized structures differed by an r.m.s. distance of 0.044 Å and a maximum distance of 0.097 Å, indicating close agreement between DFT-minimized structures even when starting from modestly different conformations.

If the unit-cell dimensions were also allowed to vary there was an anisotropic contraction, with cell axes reducing by between 1% and 3.5%, accompanied by an increase in the monoclinic angle from 90.30 (1)° to 91.8°. DFT calculations are carried out on a structure at 0 K. It is not evident whether the extent of this unit cell contraction reflects what the lattice parameters actually are at 0 K (not having measured the diffraction pattern at very low temperature), or whether it reveals shortcomings in the DFT calculation. Whatever the balance between these, the effect on the conformation of the molecule is slight, but nevertheless results in a net improvement in the agreement between the single-crystal and DFT-minimized molecules to an r.m.s. distance of 0.026 Å (maximum 0.062 Å), Table S2. This represents the best agreement between the single-crystal and DFT-D-minimized molecules starting from the fully straightened conformation.

For the powder-derived structures therefore, the C—C—C—C torsion angles were reset to 180° and the same DFT-D calculations performed, including optimization of the unit-cell parameters. The DFT-optimized molecule was taken as a rigid body and a final fine tune of the structure performed *via* the Rietveld method with *TOPAS* by a rigid-body optimization (3 rotations and 2 translations), as returning the lattice parameters from the DFT-minimized values (at 0 K) to the powder-diffraction values (at 80 or 100 K) moves the symmetry-related molecules slightly in space with respect to each other. The rigid-body refinement allows compensation for this. The powder diffraction peaks were described with the Voigt function. Also included in the fit were the scale factor, three lattice parameters, 2θ zero point correction, background parameters, a full-axial model for peak asymmetry due to axial divergence, a Stephens (1999 ▸) microstrain model for anisotropic peak broadening for all patterns except 9Me-C_29_H_59_ (which exhibited significant isotropic microstrain broadening), an overall isotropic atomic displacement parameter (*B_iso_*), and a March–Dollase correction (Dollase, 1986 ▸) for preferred orientation along 100 or 001 for the type-2 structures (*b* ≃ 4.93 Å) except 9-MeC_25_H_51_ for which, like type-1 structures, no correction was needed.

## 4. Results   

### 4.1. Single crystal   

**Figure 1:**
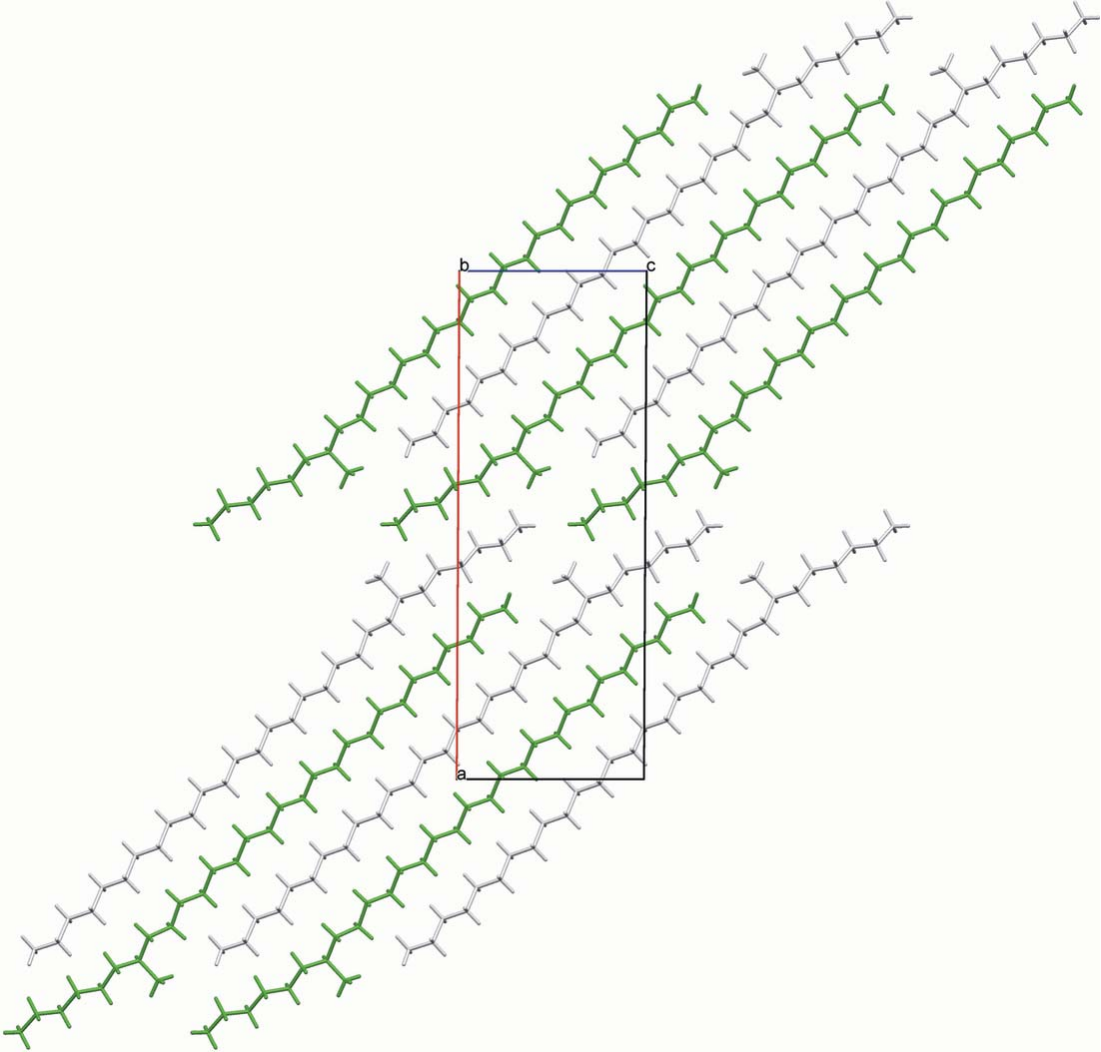
View along [010] of the structure of (*S*)-7-methylnonacosane from the single-crystal diffraction study. The grey- and green-coloured molecules are related by a 2_1_ screw axis.

**Figure 2:**
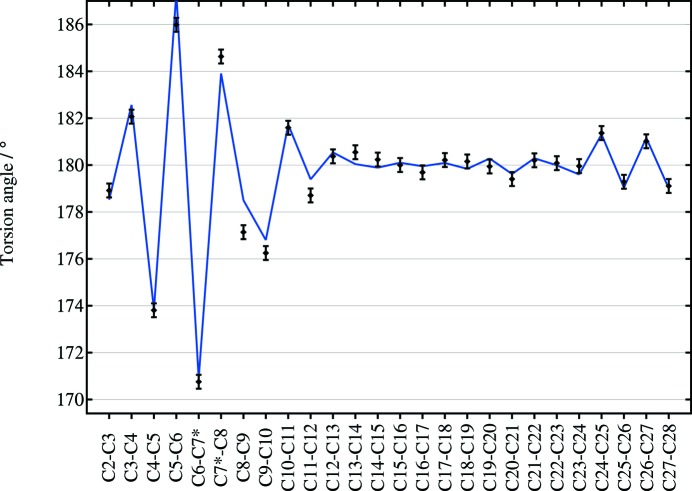
(Points with error bars) C—C—C—C torsion angles along the carbon chain of 7-methylnonacosane from the single-crystal study (standard uncertainty: 0.3°); (line) the torsion angles from the DFT-D minimized structure, starting from a straightened molecule. The horizontal axis labels the two central C atoms of the torsion. C7, to which the methyl group is attached, is emphasized with *.

The crystal structure of (*S*)-7-methylnonacosane is shown in Fig. 1 ▸. The molecule is close to straight, but has a distinct curve around the position of the methyl side chain resulting from changes in the carbon-chain torsion angles by up to 9.2 (3)° away from 180°, illustrated in Fig. 2 ▸. Also shown are the torsion angles for the DFT-optimized molecule starting from the straightened configuration, showing the correspondence between the values. The DFT calculations appear to underestimate somewhat the deviations from 180° near the side group on the longer-chain side of the molecule. It is not clear what causes this, but nevertheless the overall agreement is acceptable, with an r.m.s. deviation between C atoms of 0.026 Å (as noted above). Note that this curving of the molecule, matching closely the crystallographic result, arises spontaneously in the DFT calculation. A full list of carbon–carbon bond distances, angles and torsions is given in supplementary Table S1.

### 4.2. Powder refinements   

**Figure 3:**
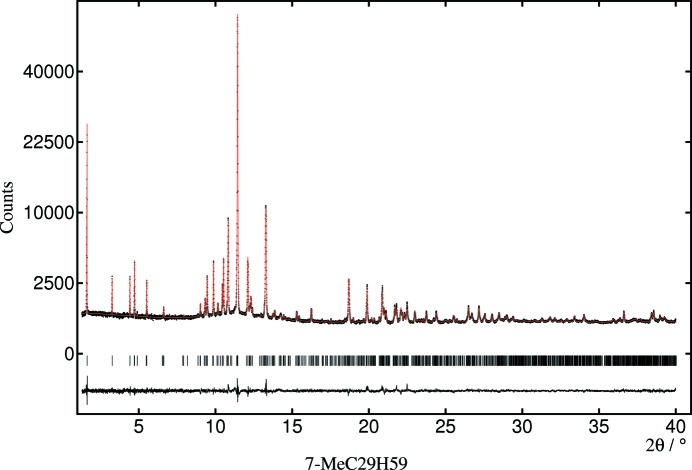
Observed, calculated and difference profiles for 7-MeC_29_H_59_ fitted with the molecule obtained from the DFT-D optimization. Note the square-root counts scale.

The results of the Rietveld fits with the DFT-optimized molecules are summarized in Table 2 ▸. The *R*-factors and plots of observed and calculated profiles indicate that the structures are consistent with the experimental powder diffraction patterns, shown for 7-MeC_29_H_59_ as an example in Fig. 3 ▸; the others are shown in the supporting information.

## 5. Discussion   

**Figure 4:**
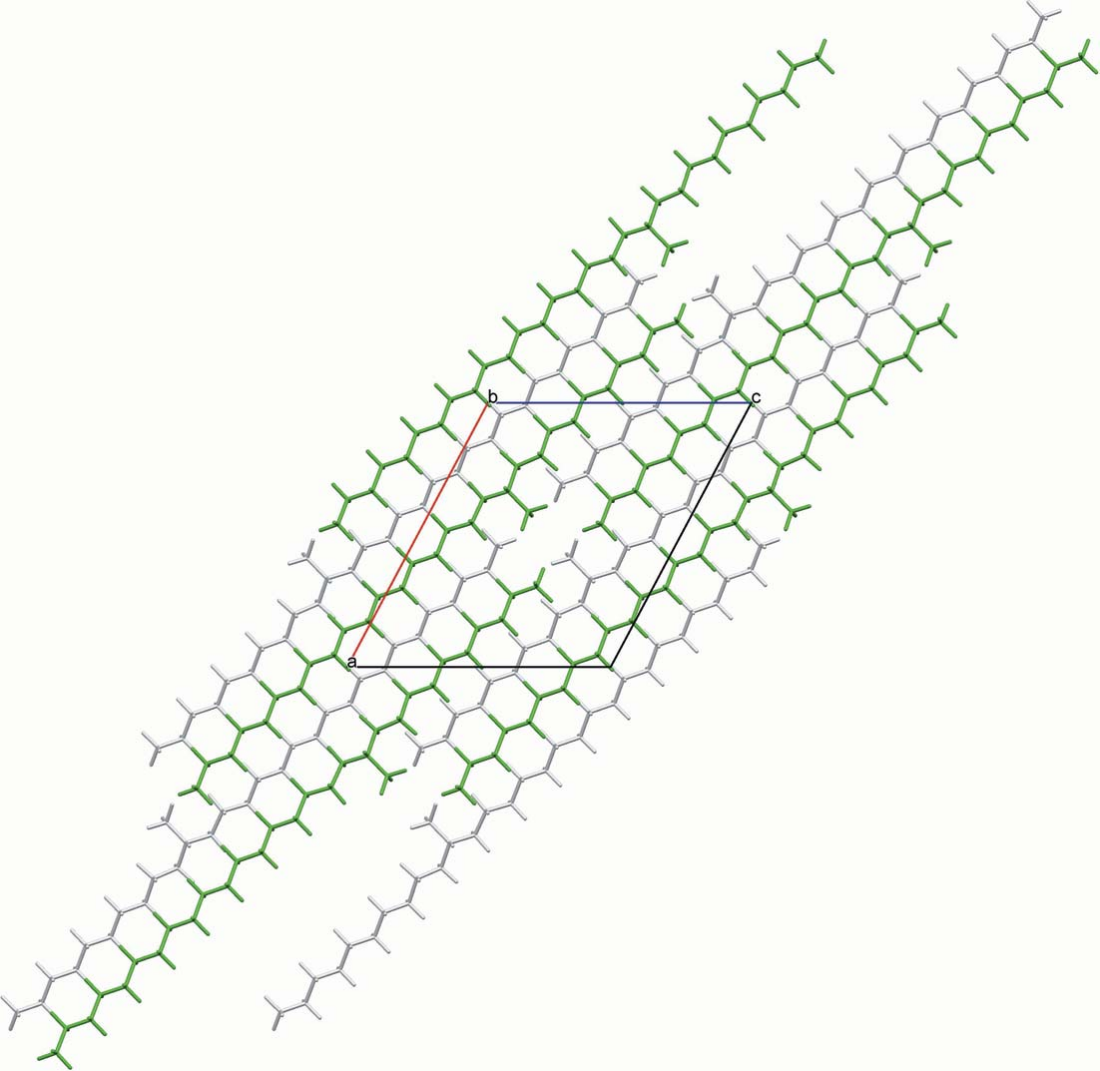
View along [010] of the structures of (*S*)-11-methylnonacosane from the DFT-D optimization.

**Figure 5:**
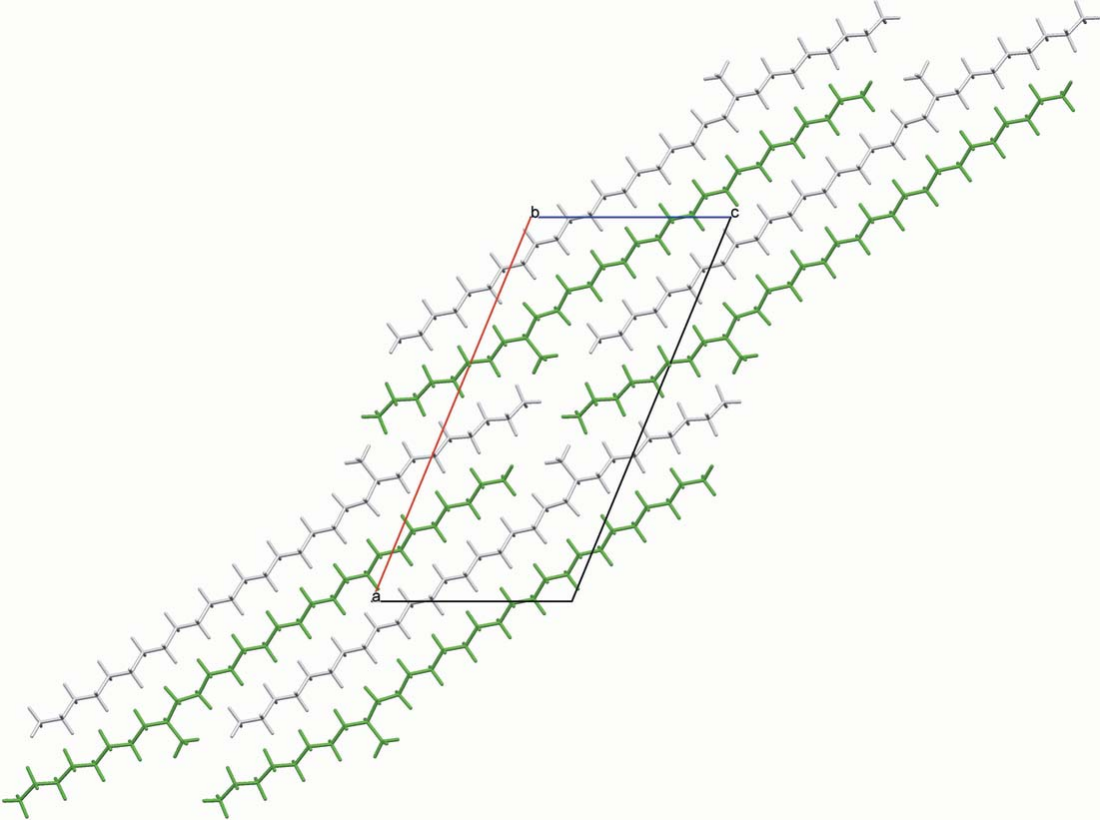
View along [010] of the structures of (*S*)-9-methylnonacosane from the DFT-D optimization.

There are two distinct packing arrangements. In the first type, corresponding to the structures with *b* ≃ 7.15 Å, the molecules are quite straight. The maximum deviation of the C—C—C—C torsion angles near the methyl group from the fully extended staggered conformation is less than 3.2°, *e.g.* see Fig. 4 ▸ illustrating the structure of 11-MeC_29_H_59_. For the second type of packing, *b* ≃ 4.93 Å, the molecules are still very much extended; however, torsion angles near the methyl group deviate more, with a maximum twist of between 8.2 and 9.1° [9.2 (3)° by single-crystal] for the molecules studied, *e.g.* 9-MeC_29_H_59_, Fig. 5 ▸, or 7-MeC_29_H_59_, Fig. 1 ▸. It appears that if the ratio of the number of C atoms in the longer and shorter sub-chains on either side of the chiral centre (C*_l_*/C*_s_*) is less than 2, packing of type 1 is observed, otherwise it is of type 2 (see Table 2 ▸). Thus, for the molecules studied here, molecules with the methyl group attached to the main chain within three C atoms of its central carbon adopt packing type 1, while those with the methyl group attached beyond four C atoms from the centre adopt packing type 2, with four C atoms representing the transition between structure types, illustrated by considering 11-MeC_29_H_59_, type 1, and 9-MeC_25_H_51_, type 2.

**Figure 6:**
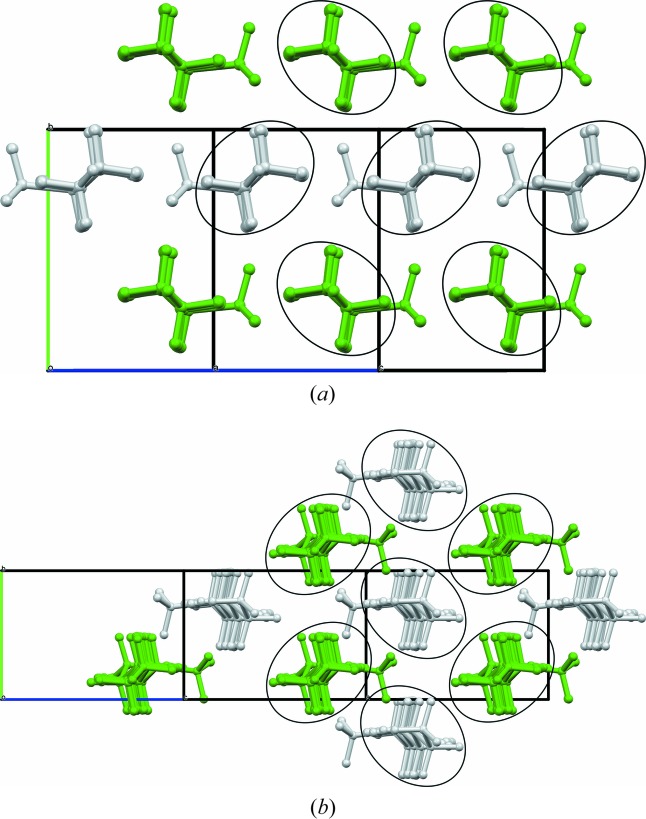
View parallel to (*a*) [102] of (*S*)-11-methylnonacosane (type 1), and (*b*) [201] of (*S*)-9-methylnonacosane (type 2), illustrating the hexagonal packing of rods. The main chain C atoms all lie close to a plane (as C—C—C—C torsion angles deviate from 180° by less than 10°) leading to distortion of the packing from perfect hexagonal, being elongated in (*a*) parallel to the *ac* plane, and in (*b*) perpendicular to the *ac* plane.

**Figure 7:**
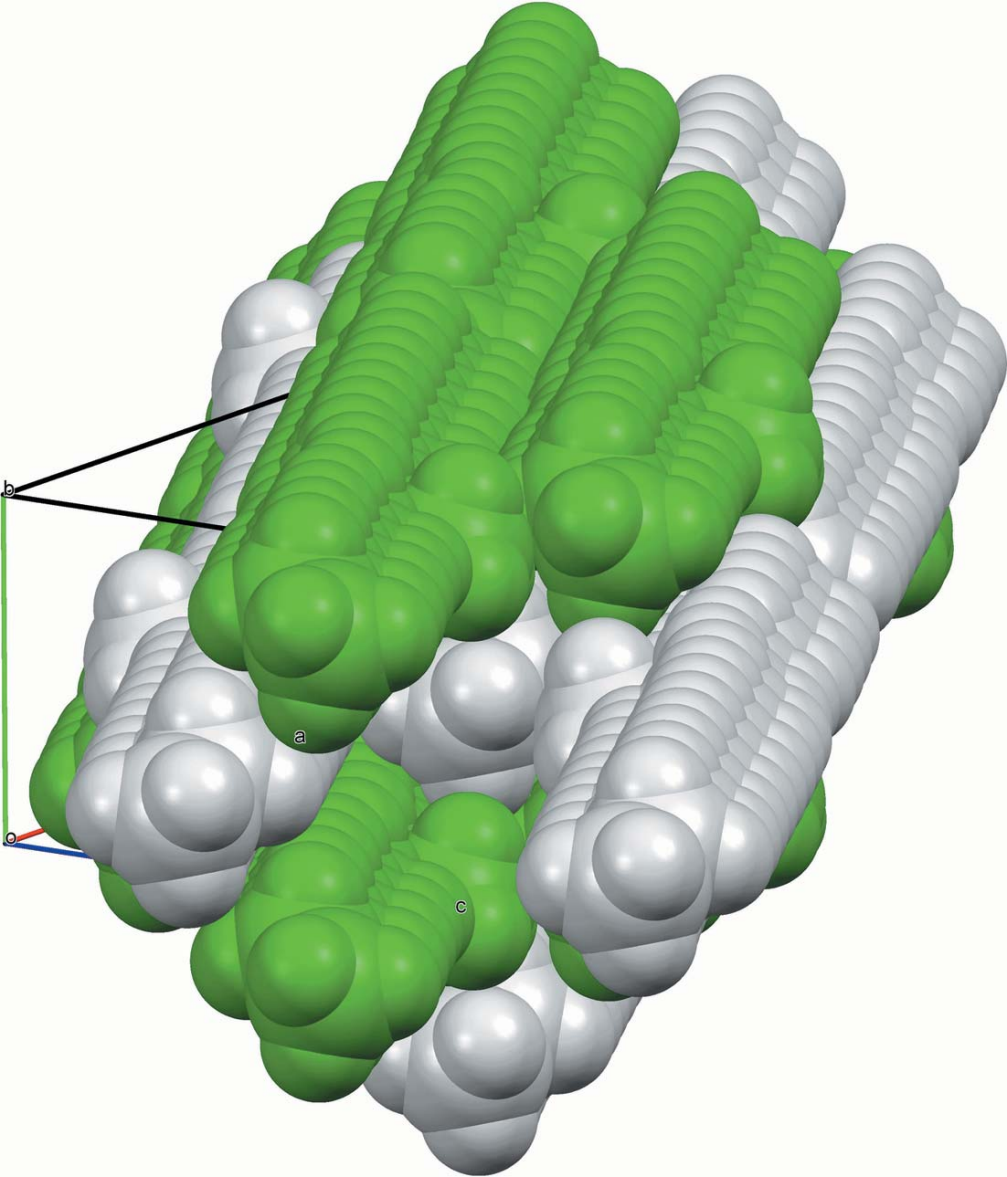
Space-filling view of the packing of 11-MeC_29_H_59_ molecules (type 1).

In both structural types, the molecules pack hexagonally like rods to maximize the stabilization afforded by the van der Waals interactions, Fig. 6 ▸. Side-chain and terminal methyl groups correlate to form distinct clusters integrated into the overall side-by-side packing. Average rod-like centre-to-centre distances of neighbouring molecules are given in Table 2 ▸. For type 1, molecules are aligned parallel to [201], positioned close to midway between (204) lattice planes. A molecule has two neighbouring molecules on either side, at the same *y* value, at an average centre-to-centre distance of *d*_102_ (4.89 − 4.91 Å for the three compounds studied) and four 2_1_-screw-related rods in layers above and below (*y* ± ½) at an average distance of ½(*d* ^2^_102_ + *b* ^2^)^1/2^ (4.33–4.34 Å). Thus the hexagonal packing of the rods is mildly elongated in one direction (parallel to the *ac* plane) with two longer and four shorter distances between centres. Similar considerations hold for the type-2 packing, in which the molecules align parallel to [102] (for the four 9-MeC_*n*_H_2*n* + 1_ structures), with molecules positioned close to midway between (402) planes. However, there are now two neighbouring molecules at ±*b* (4.93–4.94 Å) with the four others at an average distance of ½(*d* ^2^_201_ + *b* ^2^)^1/2^ (4.25–4.28 Å). Thus, with respect to the monoclinic *b* axis, the elongation of the hexagonal packing arrangement is rotated by 90° compared with type 1. When considering the hexagon of neighbouring molecules surrounding a central rod, in type 1 packing the methyl group is pointing towards the vertex of a hexagon and is parallel to the direction of elongation. In type 2 packing, the methyl group is pointing towards an edge and is perpendicular to the direction of elongation of the hexagon. In both packing types, the C–methyl bond lies close to parallel to the *ac* plane. Figs. 7 ▸ and 8 ▸ show space-filling views of the packing of the molecules for both structural types. For the single-crystal structure of 7-MeC_29_H_59_ the molecules in the chosen setting of the unit cell, with the least-obtuse β angle, are aligned parallel to [103] [so molecules are midway between (602) planes], but the unit cell can be transformed to be more easily comparable with the other type-2 structures *via* 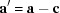 followed by a translation of the molecules by ½ 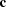.

**Figure 9:**
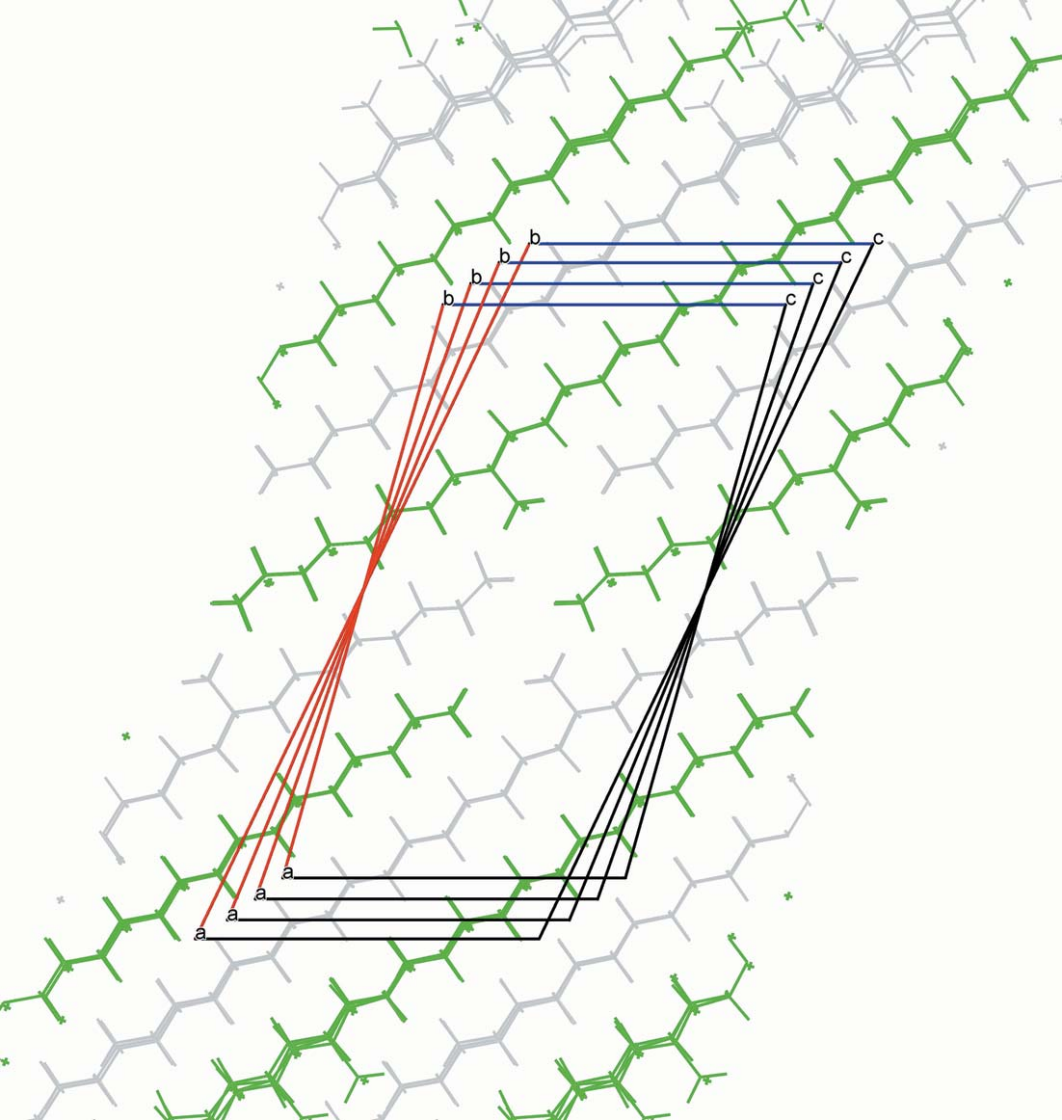
Superposition of the four 9-MeC_*n*_H_2*n* + 1_ (*n* = 25, 27, 29, 31) type-2 structures showing the reproducibility of the packing of the side and terminal methyl groups and the evolution of the unit cell with increasing chain length.

**Figure 10:**
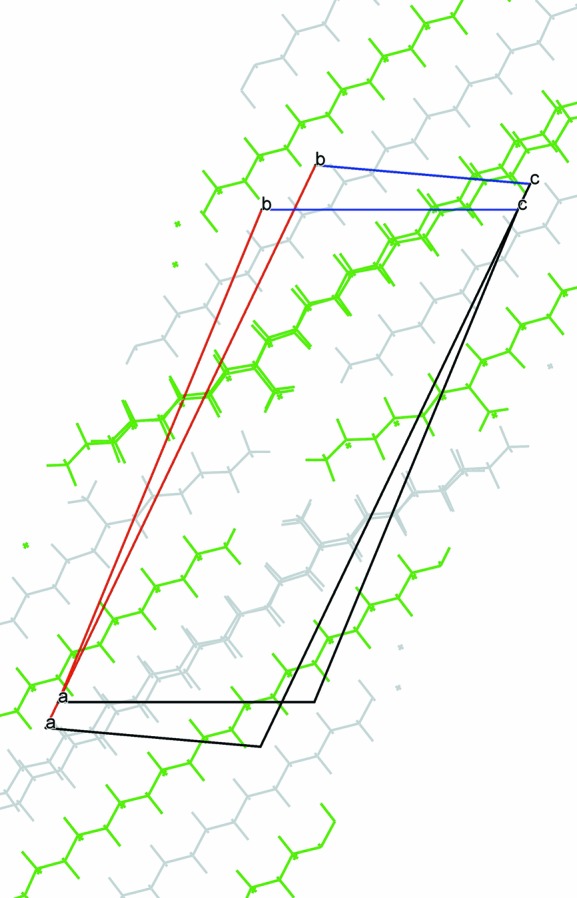
Superposition of 9-MeC_29_H_59_ and 7-MeC_29_H_59_. For the latter, the cell derived from the single-crystal study has been transformed (*via* 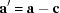, followed by a translation of the molecules by ½ 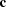) then rotated by about 4° to overlay the molecules.

**Figure 11:**
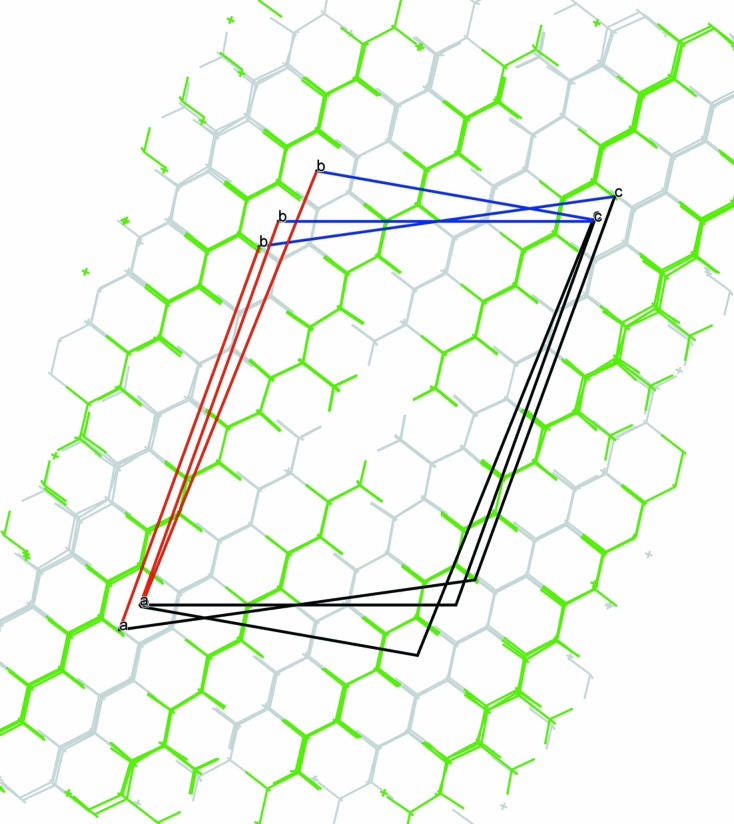
Superposition of the three type-1 structures, 11-MeC_27_H_55_, 11-MeC_29_H_59_ and 13-MeC_29_H_59_.

Each structure type has a distinct cluster of side and terminal methyl groups integrated into the overall side-by-side molecular packing scheme that is closely reproduced by the members of each series of molecules. Fig. 9 ▸ shows the superposition of the four 9-MeC_*n*_H_2*n* + 1_ type-2 structures. The length of the *c* axis increases by less than 0.4% between 9-MeC_25_H_51_ and 9-MeC_31_H_63_; the increasing length of the molecules is accommodated by increasing *a* and β. Fig. 10 ▸ shows the superposition of 9-MeC_29_H_59_ and 7-MeC_29_H_59_ which gives an idea about the effect of the methyl group position while maintaining the overall chain length. Fig. 11 ▸ shows the overlay of the type-1 structures. Again the close resemblance of the packing of the methyl groups for each substance can be seen.

## 6. Conclusion   

Two molecular packing schemes are seen for the eight molecules studied here, depending on the position of the methyl side group on the main carbon chain. If the methyl group is towards the centre of the molecule, so that the ratio of the lengths of the two carbon chains on either side of the chiral centre is less than 2, type 1 packing is observed, otherwise type 2 packing is found. The molecules pack together hexagonally as extended rods with deviations of the carbon-chain torsion angles of less than 10° from the fully extended conformation. Type-2 structures show a definite curve of the chain near the position of the methyl side group. The side-branch and terminal methyl groups of adjacent molecules are arranged together to form distinct clusters that are accommodated within the overall packing of the molecules.

The use of DFT-D optimization of the structures, following the overall approach of van de Streek & Neumann (2010 ▸, 2014 ▸), provides a consistent and well defined method to optimize the crystal structures, and avoids the large number of stereochemical restraints that would be necessary to obtain a chemically acceptable structure from the powder-diffraction data alone. Comparing the molecule obtained from the single-crystal study with that obtained from the DFT-D minimization shows good r.m.s. distance agreement between the two, and comparison with the powder diffraction pattern gives very similar fits. Fitting the DFT-D minimized molecule to the powder pattern yields an *R*_wp_ of 0.0474 (Table 2 ▸), while taking the fixed single-crystal atomic coordinates and displacement parameters yields a similar fit, with an *R*_wp_ of 0.0502, which reduces to 0.0488 if an overall isotropic temperature factor is refined in place of fixed single-crystal values, as for the DFT-optimized model.

Although stereochemical restraints should be reliable for bond distances and angles in the powder diffraction refinements, there is no obvious information on what the carbon-chain torsion angles should be for these compounds, and indeed the single-crystal derived structure showed significant deviations from perfectly staggered conformations, Fig. 2 ▸. Applying a generalized torsion restraint soft enough to allow the largest deviations from the perfectly staggered conformation is not rigid enough to hold in check the rest of the chain so that restraints would need to be specifically tailored along the chain, requiring essentially that the arrangement be known in advance. In contrast, the DFT-D approach reproduces the curved molecule for type-2 structures, and a straighter form for type-1 structures, in a chemically credible way, and yields calculated powder diffraction patterns that match those measured experimentally.

## Supplementary Material

Crystal structure: contains datablock(s) I, 13MeC29H59, 11MeC27H55, 11MeC29H59, 9MeC25H51, 9MeC27H55, 9MeC29H59, 9MeC31H63, 7MeC29H59. DOI: 10.1107/S2052252515010271/lc5062sup1.cif

Structure factors: contains datablock(s) C29Me7_SX. DOI: 10.1107/S2052252515010271/lc5062Isup2.hkl

Rietveld powder data: contains datablock(s) 13MeC29H59. DOI: 10.1107/S2052252515010271/lc506213MeC29H59sup3.rtv

Rietveld powder data: contains datablock(s) 11MeC27H55. DOI: 10.1107/S2052252515010271/lc506211MeC27H55sup4.rtv

Rietveld powder data: contains datablock(s) 11MeC29H59. DOI: 10.1107/S2052252515010271/lc506211MeC29H59sup5.rtv

Rietveld powder data: contains datablock(s) 9MeC25H51. DOI: 10.1107/S2052252515010271/lc50629MeC25H51sup6.rtv

Rietveld powder data: contains datablock(s) 9MeC27H55. DOI: 10.1107/S2052252515010271/lc50629MeC27H55sup7.rtv

Rietveld powder data: contains datablock(s) 9MeC29H59. DOI: 10.1107/S2052252515010271/lc50629MeC29H59sup8.rtv

Rietveld powder data: contains datablock(s) 9MeC31H63. DOI: 10.1107/S2052252515010271/lc50629MeC31H63sup9.rtv

Rietveld powder data: contains datablock(s) 7MeC29H59. DOI: 10.1107/S2052252515010271/lc50627MeC29H59sup10.rtv

Bond distances and angles etc. for 7-MeC29H59 from the single-crystal refinement, agreement between DFT-D and experimental 7-MeC29H59 molecules, final Rietveld profiles, and diagrams of the structures. DOI: 10.1107/S2052252515010271/lc5062sup11.pdf

CCDC references: 1403671, 1407149, 1407150, 1407151, 1407152, 1407153, 1407154, 1407155, 1407156

## Figures and Tables

**Figure 8:**
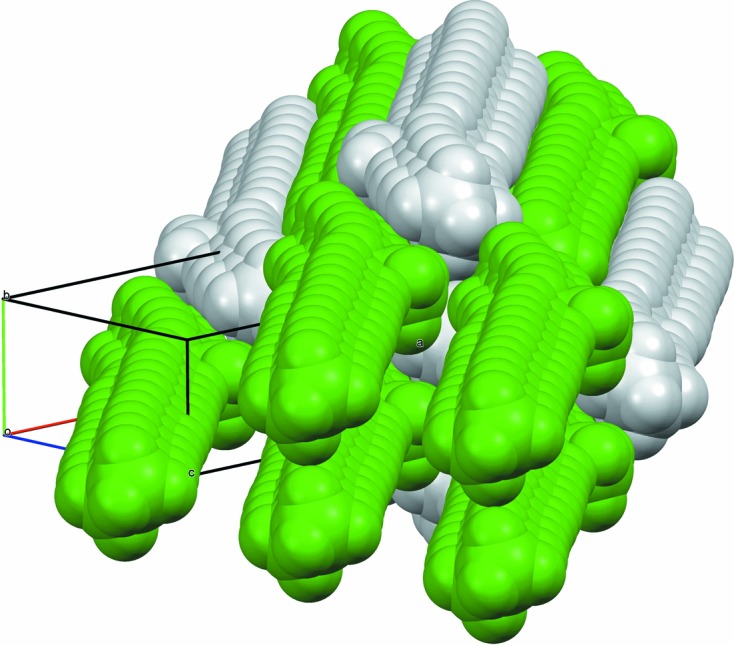
Space-filling view of the packing of 9-MeC_29_H_59_ molecules (type 2).

**Table 1:** Experimental details for single-crystal measurement on (*S*)-7-methylnonacosane

Crystal data	
Chemical formula	C_30_H_62_
*M*_r_	422.81
Crystal system, space group	Monoclinic, *P*2_1_
Temperature (K)	120
*a*, *b*, *c* ()	28.172(5), 4.935(1), 10.375(2)
()	90.30(1)
*V* (^3^)	1442.4(5)
*Z*	2
Radiation type	Synchrotron, = 0.82
(mm^1^)	0.05
Crystal size (mm)	0.2 0.2 0.02

Data collection	
Diffractometer	MAR 345 image plate BM01A (SwissNorwegian) beamline at ESRF
Absorption correction	
No. of measured, independent and observed [*I* > 2.0(*I*)] reflections	7987, 1679, 1662
*R*_int_	0.030
_max_ ()	24.3
(sin /)_max_ (^1^)	0.502

Refinement	
*R*[*F* ^2^ > 2(*F* ^2^)], *wR*(*F* ^2^), *S*	0.085, 0.080, 0.93
No. of reflections	1657
No. of parameters	121
No. of restraints	1
H-atom treatment	Riding model
_max_, _min_ (e ^3^)	0.26, 0.33

Computer programs: *CrysAlis* (Oxford Diffraction, 2006 ▸), *SIR*92 (Altomare *et al.*, 1994 ▸), *CRYSTALS* (Betteridge *et al.*, 2003 ▸).

**Table 2:** Summary of the Rietveld fits of the DFT-optimized molecules to the powder diffraction patterns Also reported are the structure type (1 or 2); the ratio of the lengths of the longer (C_l_) and shorter (C_s_) carbon chain either side of the chiral centre; and the average distances between hexagonally packed rod-like molecules (*h*0*l* = 201 for type 1 and 102 for type 2).

	13-MeC_29_H_59_	11-MeC_27_H_55_	11-MeC_29_H_59_	9-MeC_25_H_51_	9-MeC_27_H_55_	9-MeC_29_H_59_	9-MeC_31_H_63_	7-MeC_29_H_59_
*T* (K)	100	100	100	80	100	100	100	100
()	0.79975	0.79975	0.80025	0.80025	0.80105	0.80105	0.80105	0.80105
*a* ()	18.4338(4)	16.0024(3)	16.0129(2)	21.4439(8)	23.4884(13)	25.6203(2)	27.7815(4)	28.0929(4)
								30.0142(4)†
*b* ()	7.17597(9)	7.15431(8)	7.14458(7)	4.93741(8)	4.9392(1)	4.9364(2)	4.93381(4)	4.93250(3)
*c* ()	11.0207(3)	12.4017(3)	14.0964(3)	12.3456(4)	12.3352(6)	12.3267(9)	12.3899(2)	10.3689(1)
()	101.974(3)	109.825(2)	118.060(1)	105.625(2)	109.295(3)	112.448(4)	115.680(1)	90.406(1)
								110.616(1)†
*V* (^3^)	1426.11(5)	1335.67(4)	1423.13(4)	1258.81(6)	1350.7(1)	1440.8(2)	1530.52(4)	1436.77(3)
(gcm^3^)	0.985	0.982	0.987	0.967	0.971	0.975	0.978	0.977
No. of peaks	1050	981	1043	978	1047	1115	1184	1102
*R*_wp_	0.0517	0.0739	0.0528	0.0412	0.0567	0.0676	0.0628	0.0474
*R*_exp_	0.0285	0.0147	0.0295	0.0282	0.0221	0.0309	0.0278	0.0334
Type	1	1	1	2	2	2	2	2
C_l_/C_s_	1.33	1.6	1.8	2	2.25	2.5	2.75	3.67
*d*_102_ or *b*	4.89	4.91	4.91	4.94	4.94	4.94	4.93	4.93
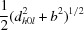	4.34	4.34	4.33	4.25	4.27	4.28	4.27	4.25

† Unit cell transformed so that 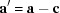 (see below and Fig. S2).
