# Supplementary material for: Crystal structures of eight mono-methyl alkanes (C26–C32) via single-crystal and powder diffraction and DFT-D optimization
Source: IUCrJ. 2015 Jul 5;2(Pt 5):490–7. doi: 10.1107/S2052252515010271 (PMC4547817; doi:10.1107/S2052252515010271)
Supplement: Supplementary file 11 [file m-02-00490-sup11.pdf]

IUCrJ

Volume 2 (2015)

Supporting information for article:

Crystal structures of eight mono-methyl alkanes (C26–C32) *via* single-crystal and powder diffraction and DFT-D optimization

Lee Brooks, Michela Brunelli, Philip Pattison, Graeme Jones and Andrew Fitch

Supporting Information

Table S1 Bond distances, angles and torsions (Å, º) for 7-methylnanacosane from the single-crystal study.

C1—C2	1.513 (5)	C15—C16	1.524 (4)
C2—C3	1.510 (5)	C16—C17	1.519 (4)
C3—C4	1.536 (5)	C17—C18	1.522 (4)
C4—C5	1.522 (5)	C18—C19	1.518 (4)
C5—C6	1.519 (5)	C19—C20	1.525 (4)
C6—C7	1.523 (5)	C20—C21	1.522 (4)
C7—C8	1.531 (5)	C21—C22	1.528 (4)
C7—C30	1.521 (4)	C22—C23	1.520 (5)
C8—C9	1.526 (5)	C23—C24	1.528 (5)
C9—C10	1.520 (5)	C24—C25	1.522 (4)
C10—C11	1.519 (4)	C25—C26	1.522 (5)
C11—C12	1.516 (4)	C26—C27	1.522 (4)
C12—C13	1.527 (4)	C27—C28	1.527 (5)
C13—C14	1.528 (4)	C28—C29	1.516 (5)
C14—C15	1.525 (4)		
C1—C2—C3	114.1 (3)	C14—C15—C16	113.9 (3)
C2—C3—C4	112.9 (3)	C15—C16—C17	114.2 (3)
C3—C4—C5	115.3 (3)	C16—C17—C18	114.4 (3)
C4—C5—C6	113.0 (3)	C17—C18—C19	114.4 (3)
C5—C6—C7	117.4 (3)	C18—C19—C20	113.9 (3)
C6—C7—C8	110.3 (3)	C19—C20—C21	114.1 (3)
C6—C7—C30	111.9 (3)	C20—C21—C22	114.0 (3)
C8—C7—C30	112.0 (3)	C21—C22—C23	113.9 (3)
C7—C8—C9	115.4 (3)	C22—C23—C24	113.9 (3)
C8—C9—C10	114.6 (3)	C23—C24—C25	114.0 (3)
C9—C10—C11	113.4 (3)	C24—C25—C26	114.4 (3)

C10—C11—C12	114.9 (3)	C25—C26—C27	114.0 (3)
C11—C12—C13	113.3 (3)	C26—C27—C28	113.8 (3)
C12—C13—C14	114.1 (3)	C27—C28—C29	113.5 (3)
C13—C14—C15	113.8 (3)		
C1—C2—C3—C4	178.9 (3)	C15—C16—C17—C18	179.7 (3)
C2—C3—C4—C5	-177.9 (3)	C16—C17—C18—C19	-179.8 (3)
C3—C4—C5—C6	173.8 (3)	C17—C18—C19—C20	-179.9 (3)
C4—C5—C6—C7	-174.0 (3)	C18—C19—C20—C21	179.9 (3)
C5—C6—C7—C8	170.8 (3)	C19—C20—C21—C22	179.4 (3)
C6—C7—C8—C9	-175.4 (3)	C20—C21—C22—C23	-179.8 (3)
C7—C8—C9—C10	177.1 (3)	C21—C22—C23—C24	-179.9 (3)
C8—C9—C10—C11	176.3 (3)	C22—C23—C24—C25	180.0 (3)
C9—C10—C11—C12	-178.4 (3)	C23—C24—C25—C26	-178.6 (3)
C10—C11—C12—C13	178.7 (3)	C24—C25—C26—C27	179.3 (3)
C11—C12—C13—C14	-179.6 (3)	C25—C26—C27—C28	-179.0 (3)
C12—C13—C14—C15	-179.5 (3)	C26—C27—C28—C29	179.1 (3)
C13—C14—C15—C16	-179.8 (3)	C5—C6—C7—C30	-63.8 (5)
C14—C15—C16—C17	180.0 (3)	C30—C7—C8—C9	59.3 (5)

Table S2 Overlay in Mercury of the single-crystal and the DFT-optimised 7-Me-$C_{29}H_{59}$ molecules

Structure Overlay

Select **pairs of atoms** in any two crystal structures.

Overlay

		**Atom1**	Atom2	Distance
1	Delete	C1	C1	0.053
2	Delete	C2	C2	0.062
3	Delete	C3	C3	0.028
4	Delete	C4	C4	0.021
5	Delete	C5	C5	0.044
6	Delete	C6	C6	0.009
7	Delete	C7	C7	0.018
8	Delete	C8	C8	0.025
9	Delete	C9	C9	0.008
10	Delete	C10	C10	0.013
11	Delete	C11	C11	0.005
12	Delete	C12	C12	0.014
13	Delete	C13	C13	0.017
14	Delete	C14	C14	0.020
15	Delete	C15	C15	0.022
16	Delete	C16	C16	0.017
17	Delete	C17	C17	0.020
18	Delete	C18	C18	0.013
19	Delete	C19	C19	0.015
20	Delete	C20	C20	0.014
21	Delete	C21	C21	0.011
22	Delete	C22	C22	0.016
23	Delete	C23	C23	0.012
24	Delete	C24	C24	0.018
25	Delete	C25	C25	0.017
26	Delete	C26	C26	0.023
27	Delete	C27	C27	0.019
28	Delete	C28	C28	0.026
29	Delete	C29	C29	0.030
30	Delete	C30	C30	0.046

RMS: **0.0256**

Close

Figure S1 Observed (points), calculated (red line) and difference profiles for the powder diffraction patterns for the eight samples after a final rigid body fit of the molecules obtained from the DFT-D optimisation. Note the square-root counts scale.

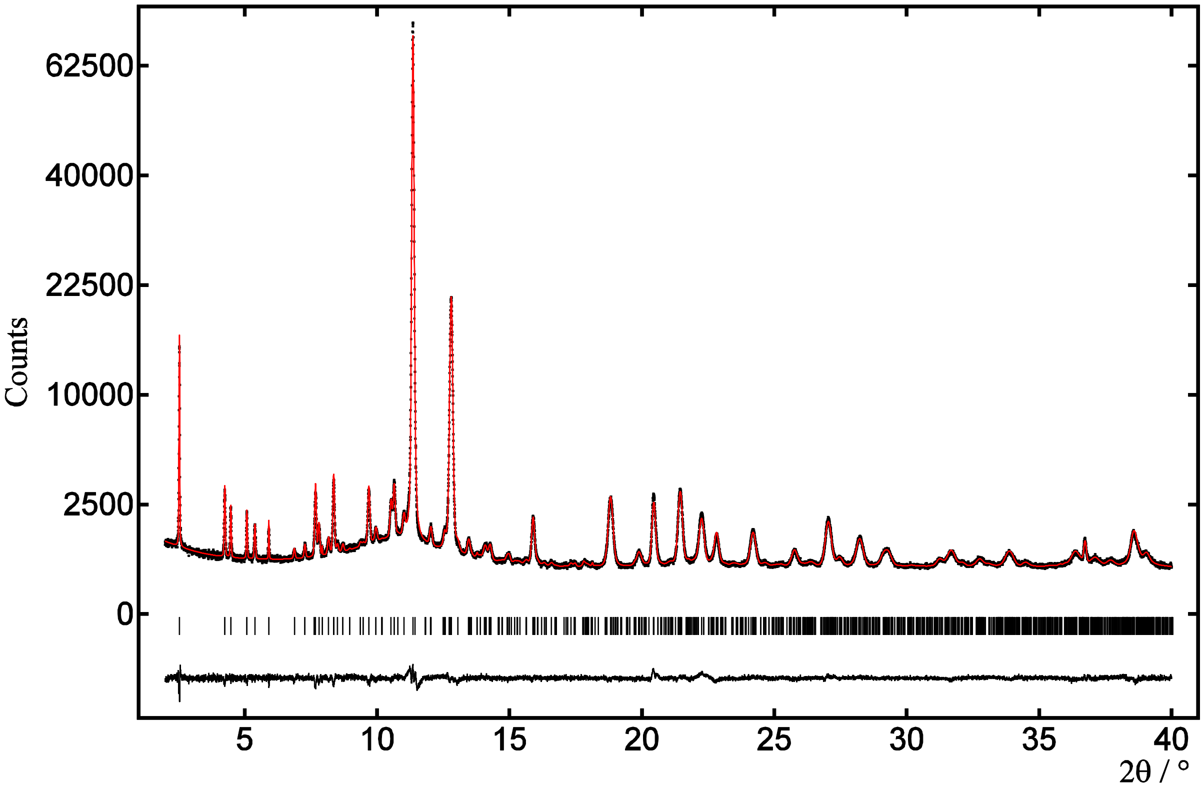

13-MeC29H59

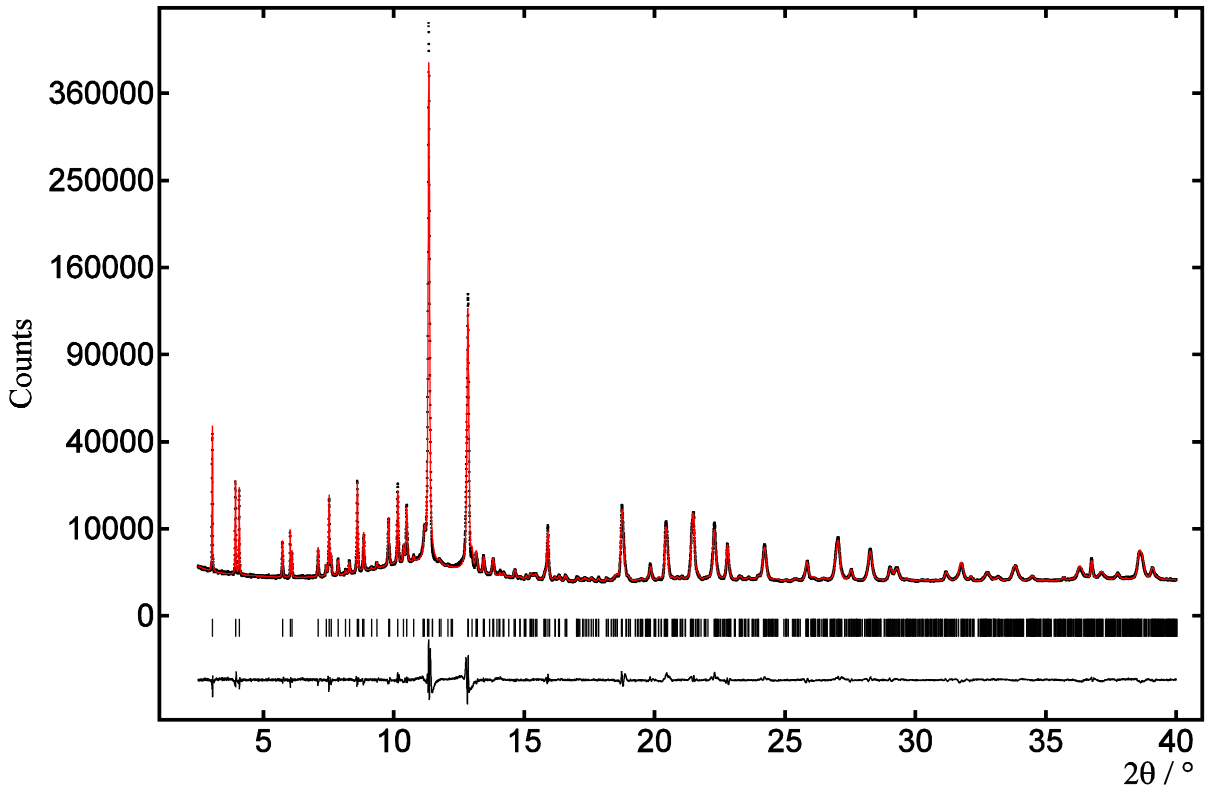

11-MeC27H55

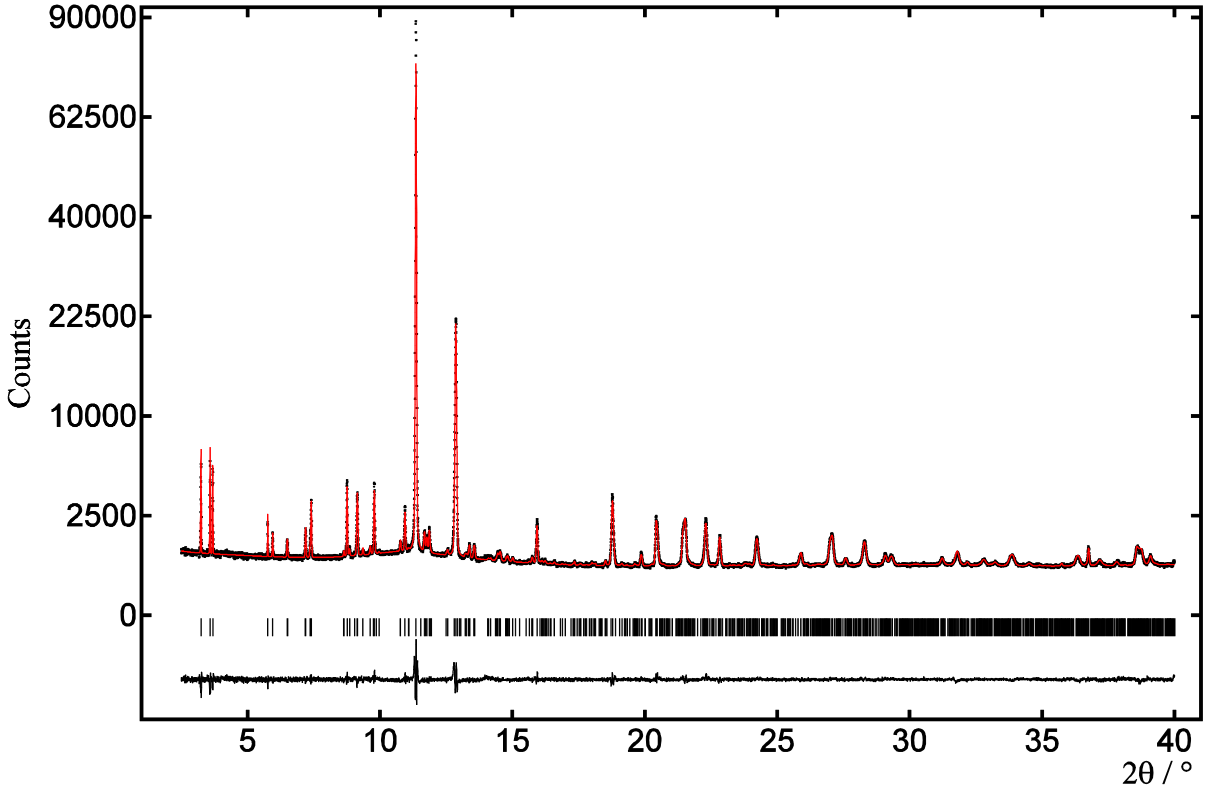

11-MeC29H59

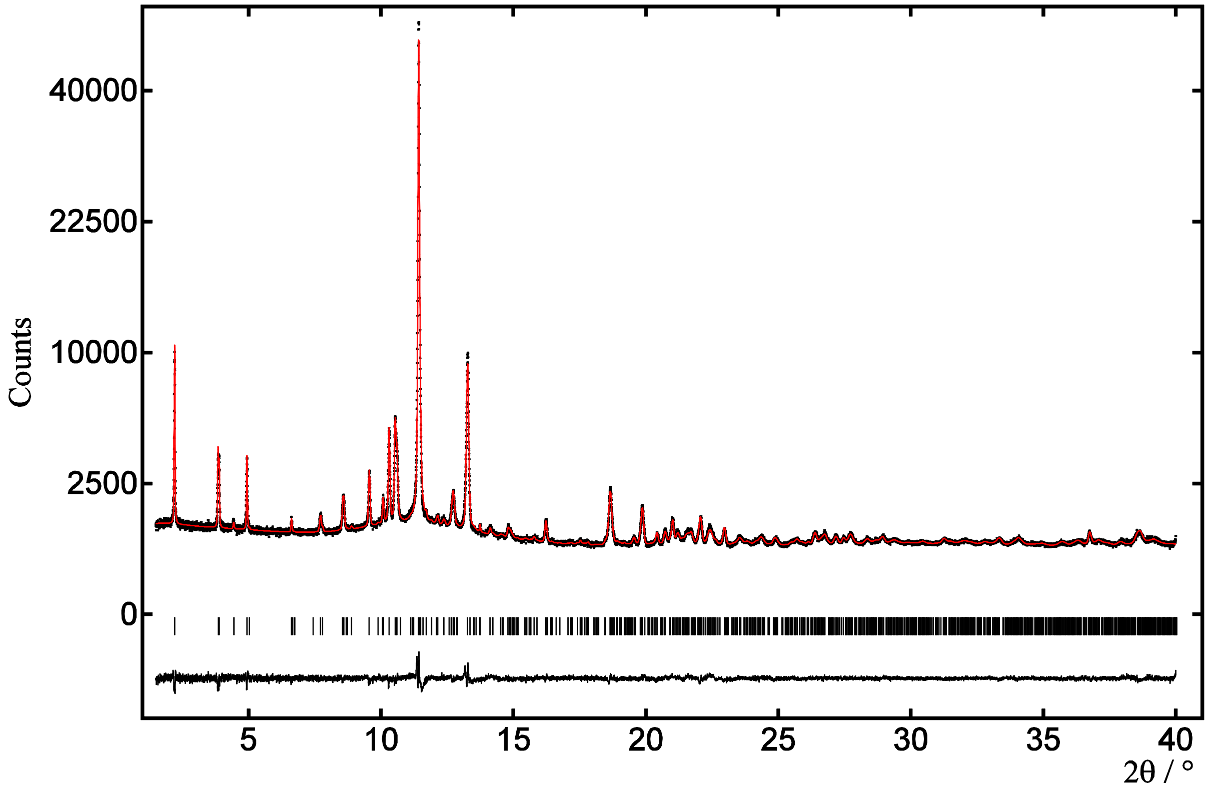

9-MeC25H51

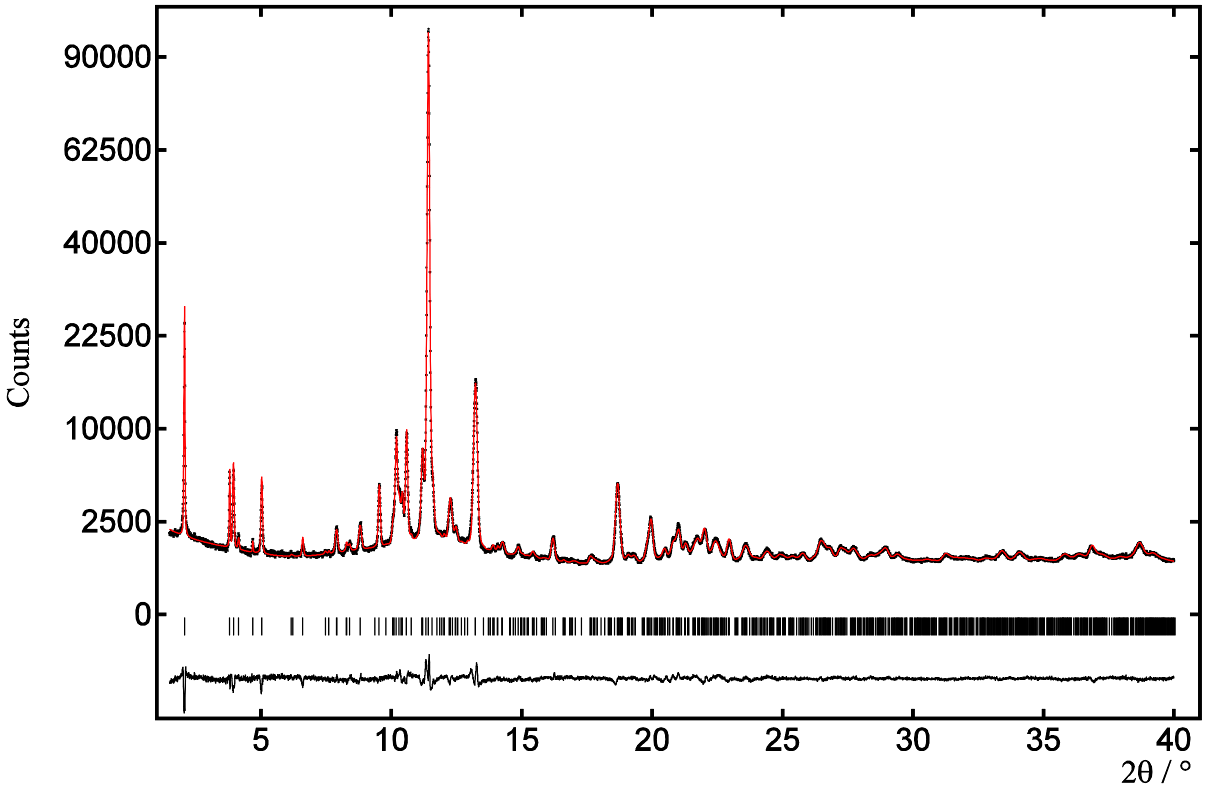

9-MeC27H55

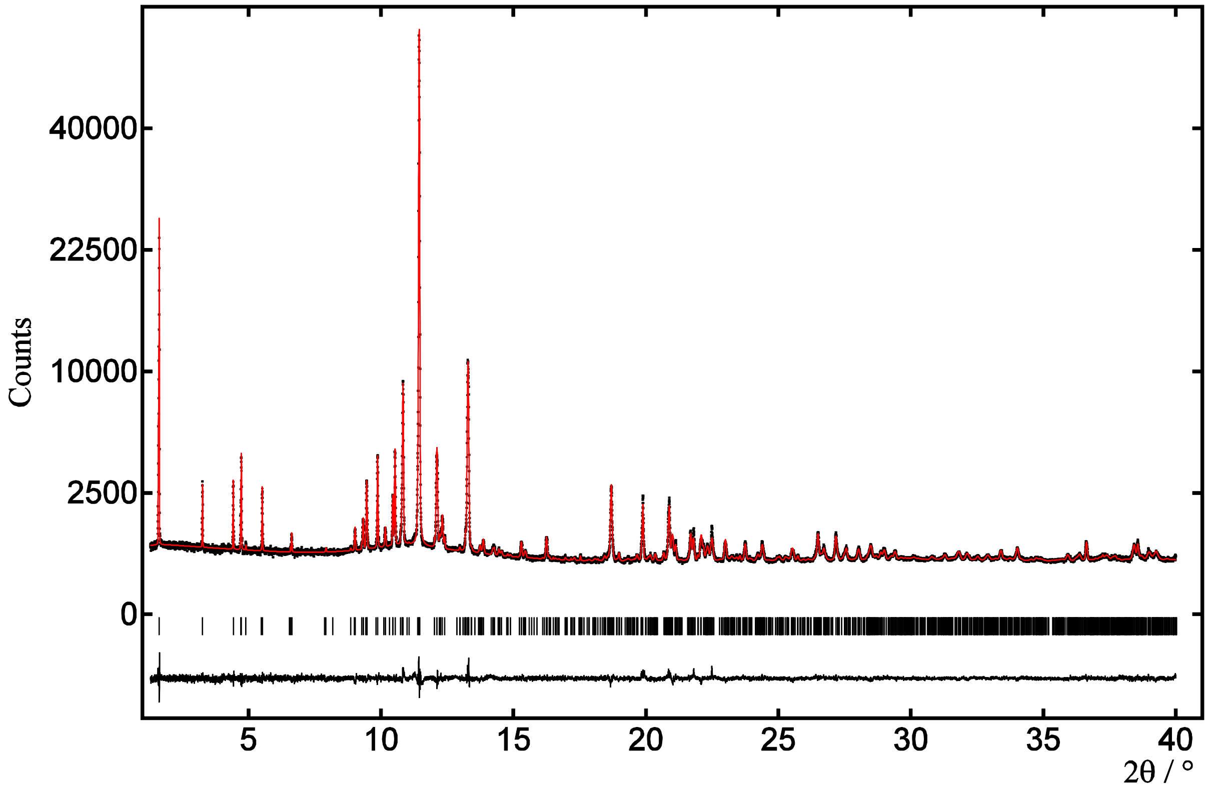

7-MeC29H59

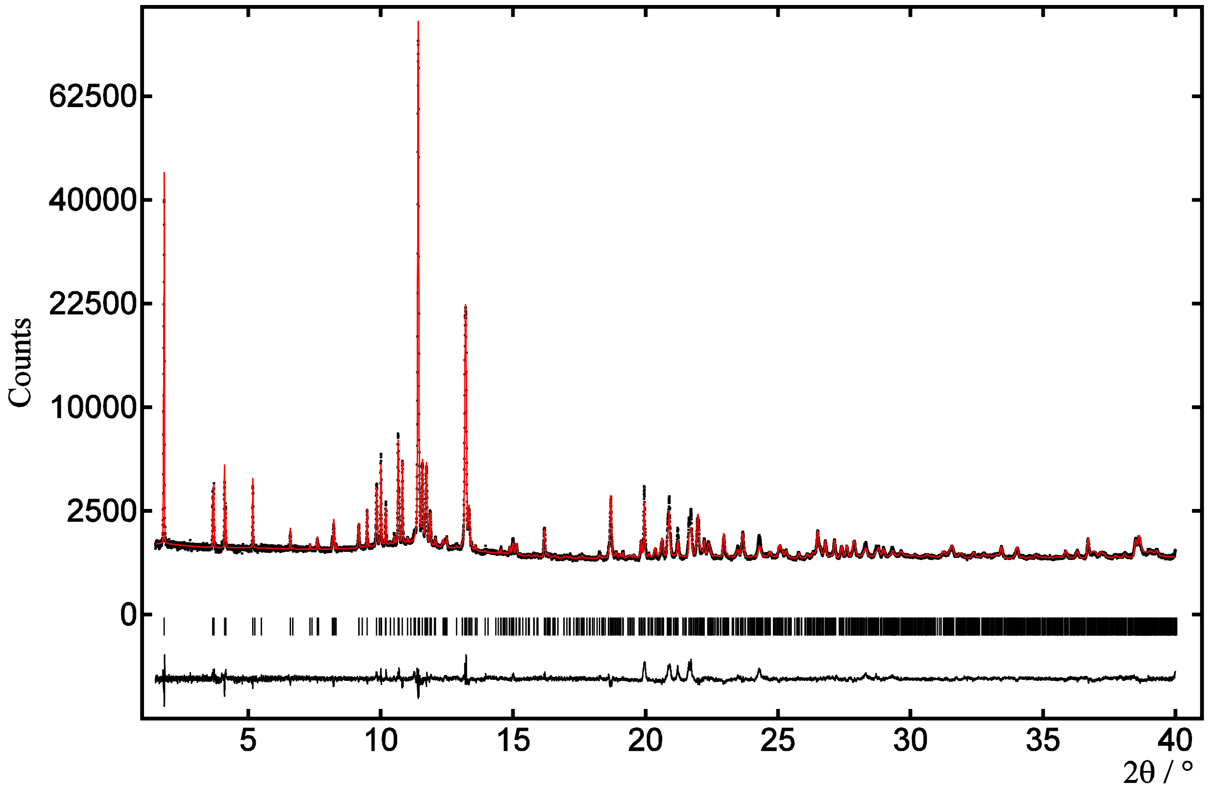

9-MeC31H63

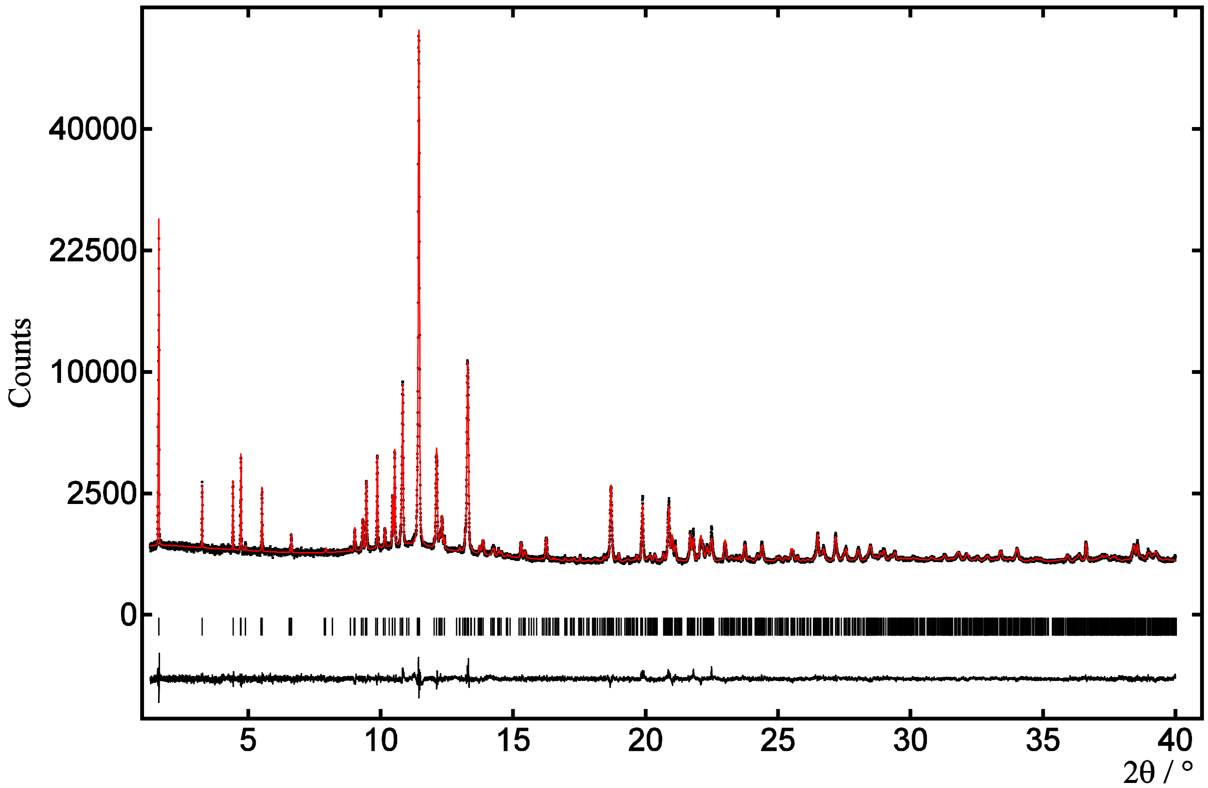

7-MeC29H59

Figure S2 The DFT-minimised structures viewed along the *b* axis. Grey and green molecules are related by a 2_1 screw axis.

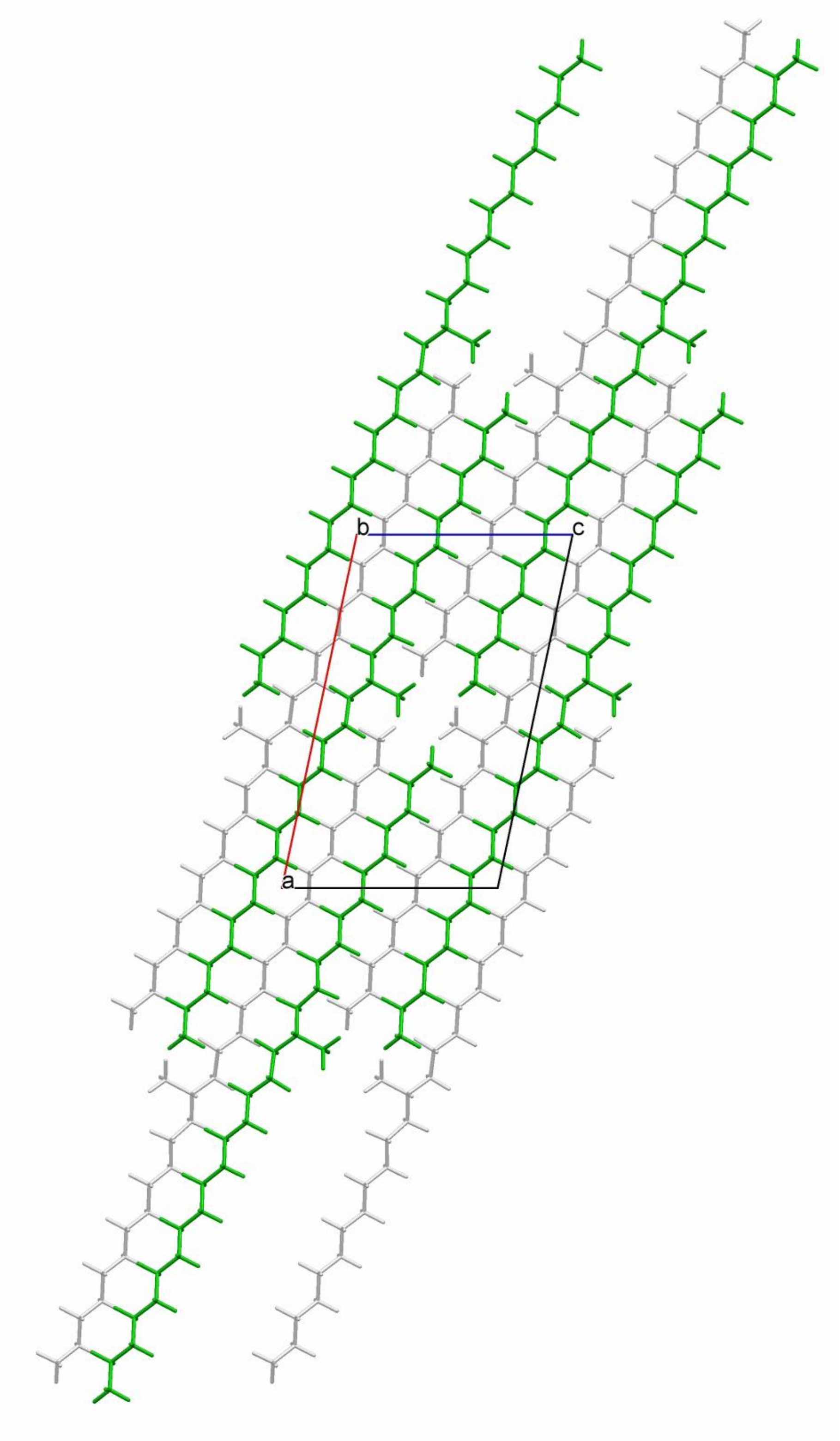

13-Me$C_{29}H_{59}$

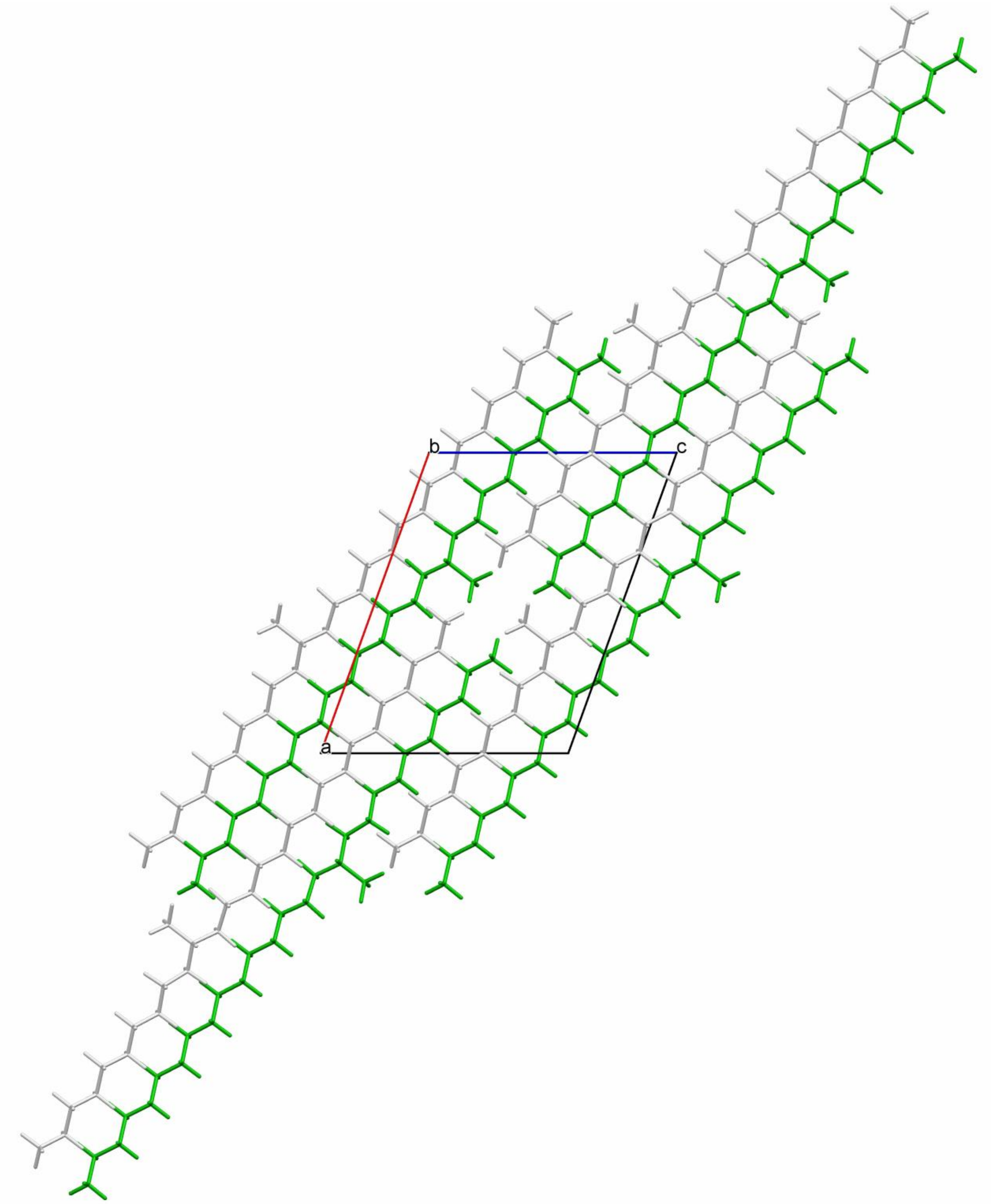

11-$MeC_{27}H_{55}$

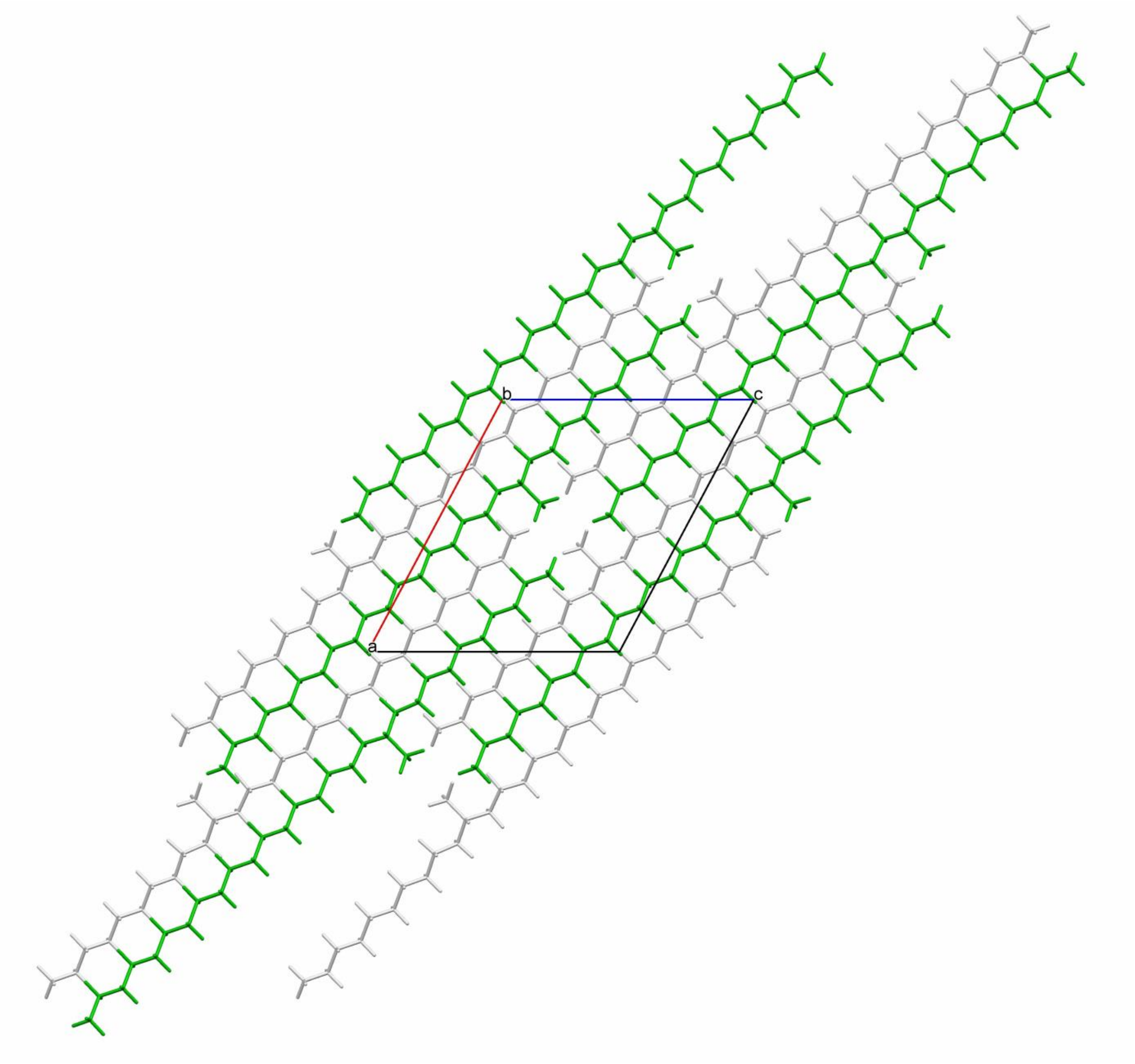

11-Me$C_{29}H_{59}$

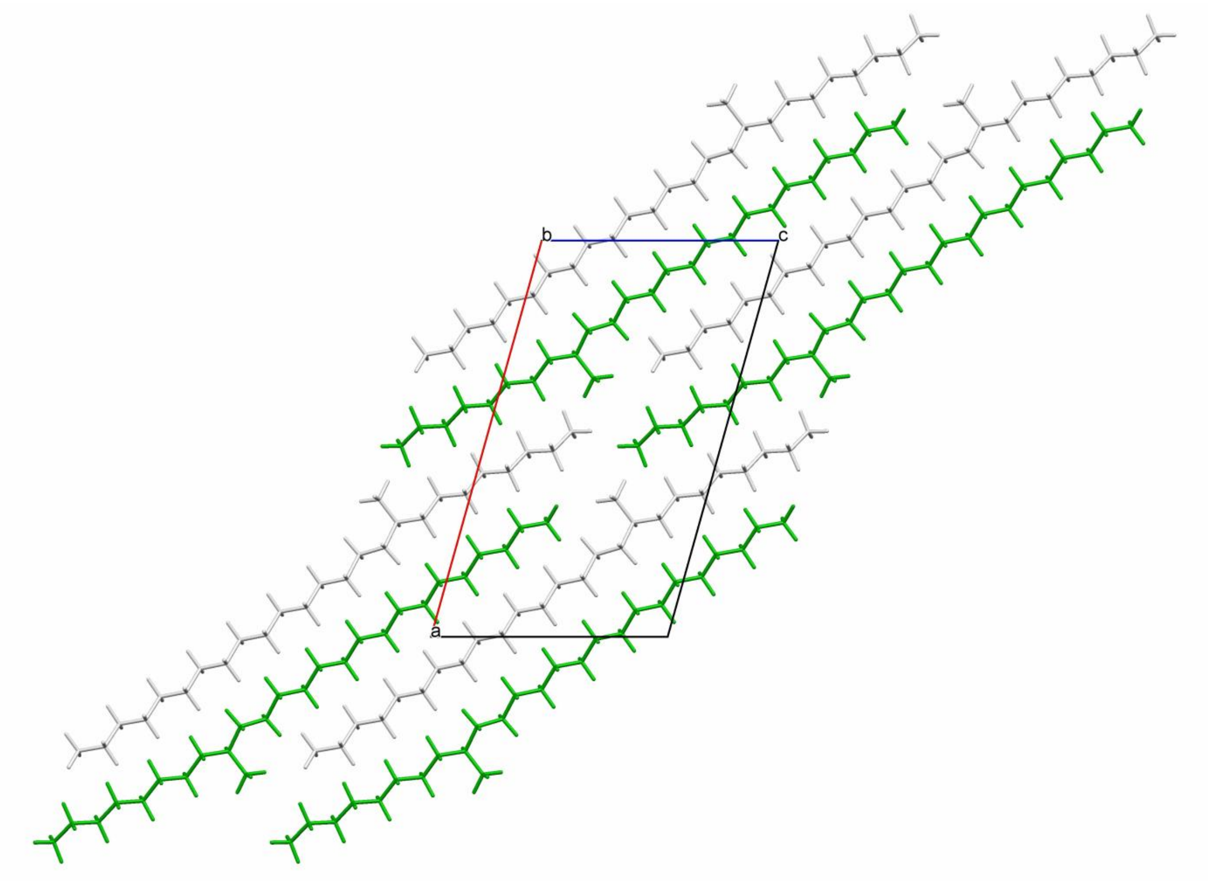

9-Me$C_{25}H_{51}$

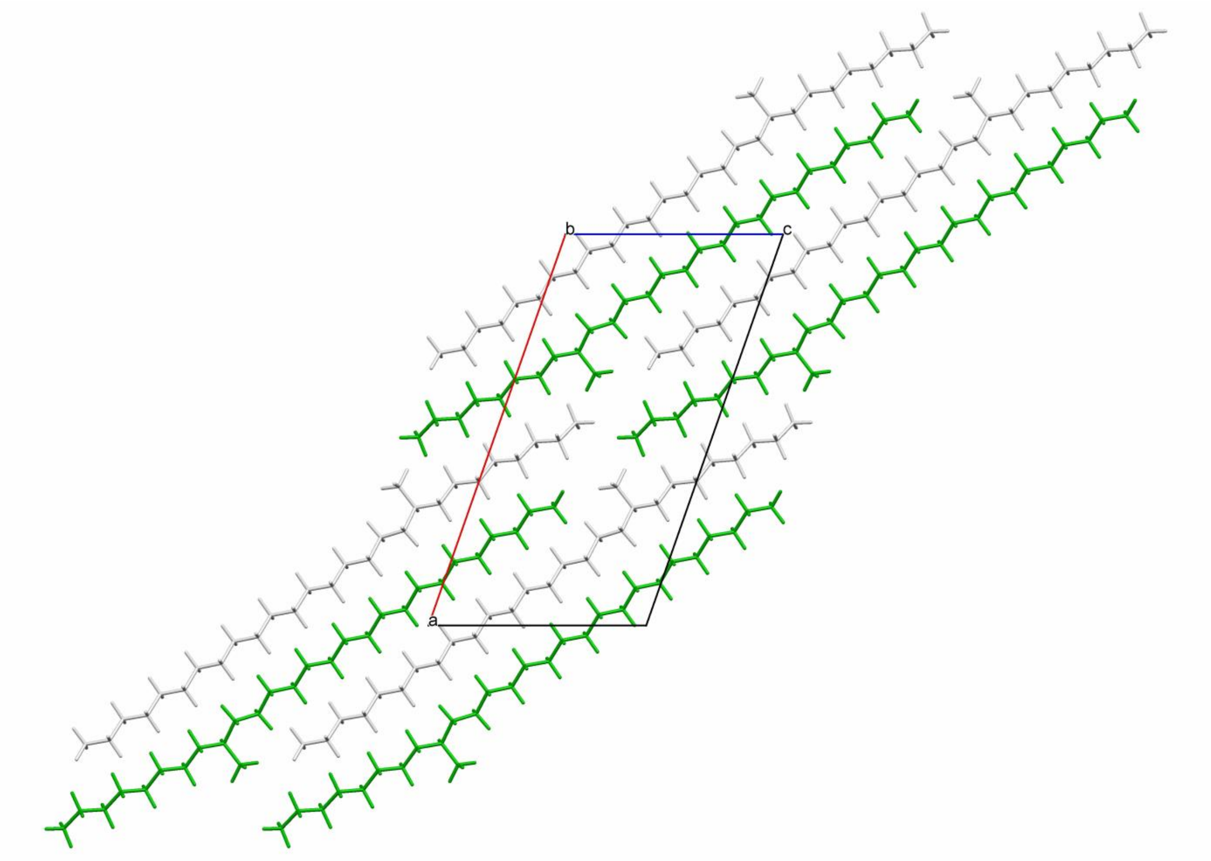

9-Me$C_{27}H_{55}$

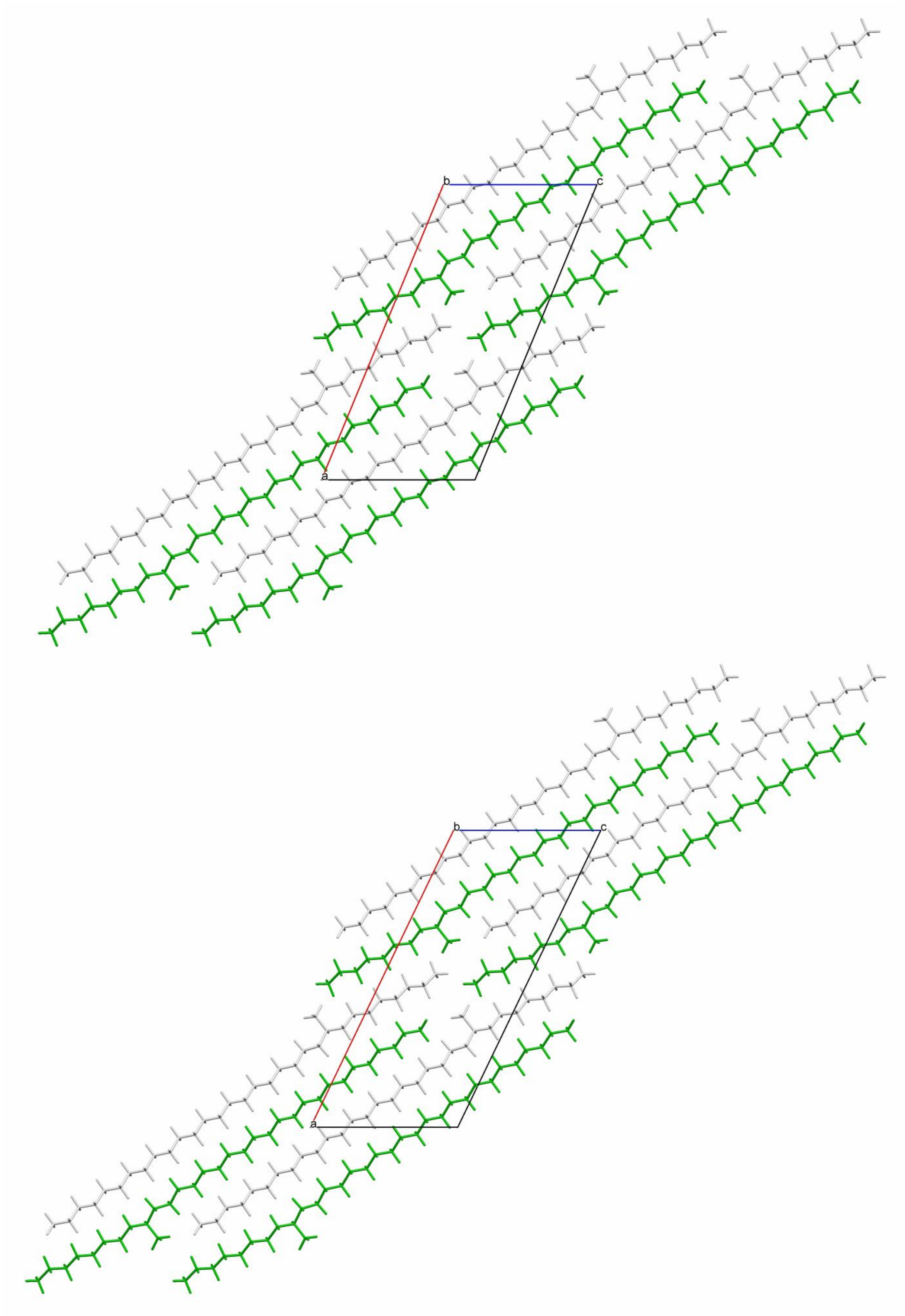

9-Me$C_{29}H_{59}$

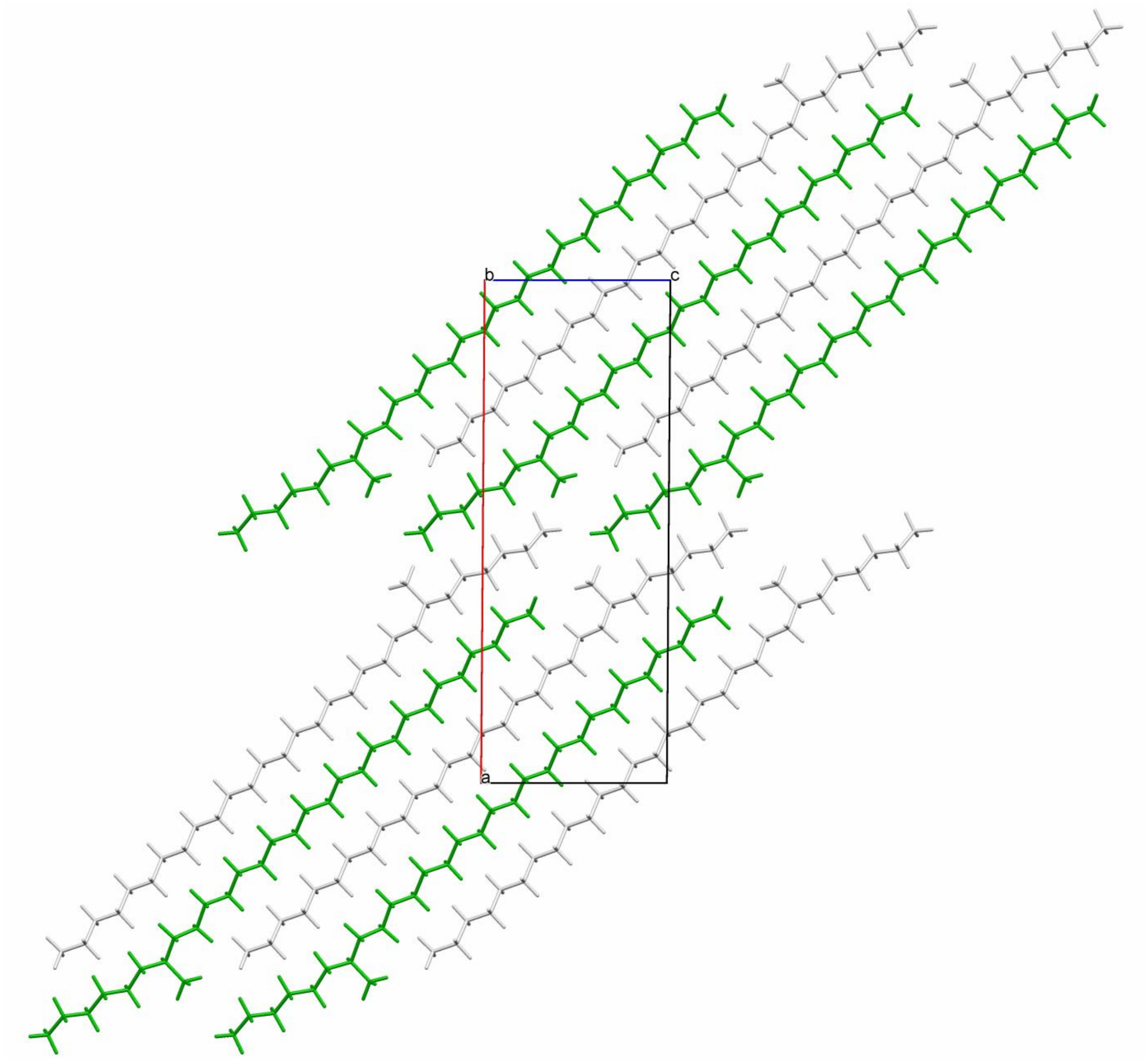

9-$MeC_{31}H_{63}$

7-$MeC_{29}H_{59}$

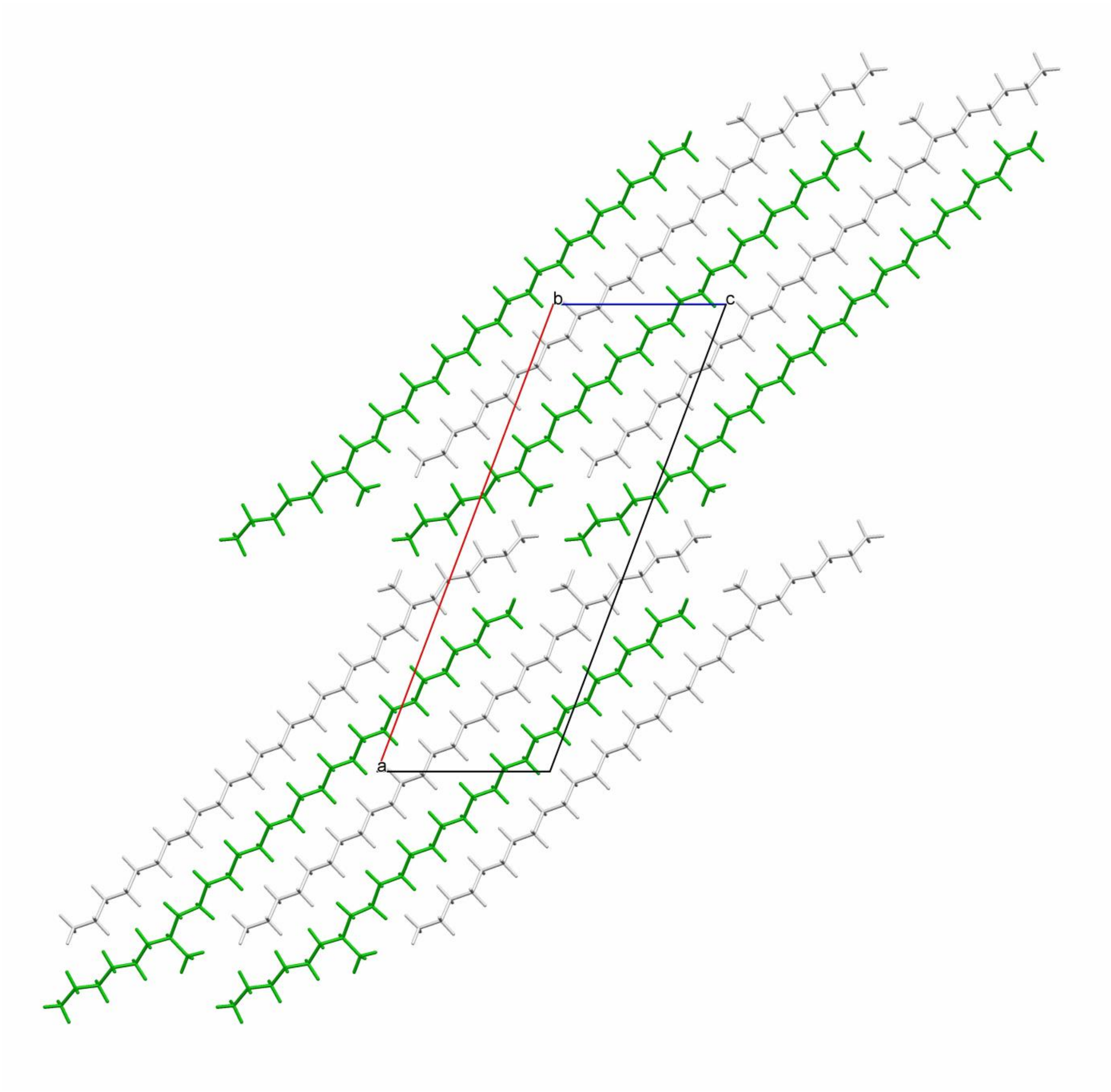

7-$MeC_{29}H_{59}$ with the unit cell transformed via $\overrightarrow{a'} = \vec{a} - \vec{c}$, followed by a translation of the molecules by ½$\vec{c}$.
